# DeepD&Cchl: an AI tool for automated 3D single-cell chloroplast detection, counting, and cell type clustering

**DOI:** 10.3389/fpls.2025.1513953

**Published:** 2025-05-23

**Authors:** Qun Su, Le Liu, Zhengsheng Hu, Tao Wang, Huaying Wang, Qiuqi Guo, Xinyi Liao, Yan Sha, Feng Li, Zhao Dong, Shaokai Yang, Ningjing Liu, Qiong Zhao

**Affiliations:** ^1^ School of Mathematics and Physics, Hebei University of Engineering, Handan, Hebei, China; ^2^ School of Life Sciences, East China Normal University, Shanghai, China; ^3^ National Satellite Meteorological Centre, Beijing, China; ^4^ University of Alberta, Edmonton, AB, Canada; ^5^ The High School Affiliated to Renmin University of China, Beijing, China; ^6^ Hebei Computational Optical Imaging and Photoelectric Detection Technology Innovation Center, Hebei University of Engineering, Handan, Hebei, China; ^7^ Department of Physics, University of Alberta, Edmonton, AB, Canada; ^8^ Institute of Eco-Chongming, Shanghai, China; ^9^ Zhejiang Zhoushan Island Ecosystem Observation and Research Station, Zhoushan, Zhejiang, China

**Keywords:** chloroplasts, DeepD&Cchl, deep learning, automatic detection and counting, single cell, cell type clustering

## Abstract

Chloroplast density in cells varies among different types of cells and plants. In current single-cell spatiotemporal analysis, the automatic detection and quantification of chloroplasts at the single-cell level is crucial. We developed DeepD&Cchl (Deep-learning-based Detecting-and-Counting-chloroplasts), an AI tool for single-cell chloroplast detection and cell-type clustering. It utilizes You-Only-Look-Once (YOLO), a real-time detection algorithm, for accurate and efficient performance. DeepD&Cchl has been proved to identify chloroplasts in plant cells across various imaging types, including light microscopy, electron microscopy, and fluorescence microscopy. Integrated with an Intersection Over Union (IOU) module, DeepD&Cchl precisely counts chloroplasts in single- or multi-layered images, while eliminating double-counting errors. Furthermore, when combined with Cellpose, a single-cell segmentation tool, DeepD&Cchl enhances its effectiveness at the single-cell level. By counting chloroplasts within individual cells, it supports cell-type-specific clustering based on chloroplast number versus cell size, offering valuable morphological insights for single-cell studies. In summary, DeepD&Cchl is a significant advancement in plant cell analysis. It offers accuracy and efficiency in chloroplast identification, counting and cell-type classification, providing a useful tool for plant research.

## 1 Introduction

Chloroplasts convert light energy to chemical energy through photosynthesis, provide oxygen, and serve as a cornerstone for the world. Research on chloroplasts primarily focuses on their material composition, genome, diversity, evolution, structure, as well as function and adaptation (Liu et al., 2018; Xing et al., 2025; Han et al., 2025; Long et al., 2024; Parisa et al., 2025). The research usually needs to quantify chloroplast numbers. Accurate counting is essential for evaluating photosynthetic efficiency and related physiological traits. Chloroplast number at the stomatal cells has been used as a reliable indicator for identifying hybrid species or estimating the ploidy level of specific plant tissues (Fujiwara et al., 2019; Watts et al., 2023; Cao et al., 2024). Studies have shown that it varies among different genetic backgrounds of the same species, suggesting its potential as a classification marker (Pyke and Leech, 1994; Pyke et al., 1994). Ukwueze et al. used chloroplast counting methods to examine the ploidy of banana germplasm (Ukwueze et al., 2022). Chepkoech et al. reported an increase in chloroplast numbers in tetraploid potatoes compared to diploid plants (Chepkoech et al., 2019). Moreover, Pyke et al. found that the average number of chloroplasts per cell in the initial leaves varies among different genetic backgrounds of Arabidopsis (Pyke and Leech, 1994; Pyke et al., 1994). Specifically, the Landsberg erecta (Ler) ecotype has 121 chloroplasts per cell, while Wassilewskija (Ws) ecotype has 83 (Pyke and Leech, 1994; Pyke et al., 1994). These findings suggest that chloroplast numbers can act as a classification marker, and counting chloroplasts in single cells is key to understanding plant adaptability, evolution, and diversity.

Building on the importance of single-cell analysis, a key question in plant developmental biology is how a plant cell differentiates into a specific type. In single-cell biology, cells are typically classified by their transcriptomes. It’s important to understand that the transcriptome acts as a snapshot of a cell’s status at a particular moment, considering that cells are dynamic and continuously adapt to signals and cell cycle changes. This necessitates the integration of subcellular morphological information, for instance, chloroplast status, to accurately define a cell type.

However, current methods have trouble in quickly and accurately counting chloroplasts at the single-cell level. Manual or software-assisted counting using images is time-consuming and error-prone. Molecular staining for organelle counting, including chloroplasts, associated with flow cytometry might seem efficient. But it is limited by the isolation process and can’t accurately determine the number per cell (Mattiasson, 2004; Cole, 2016). A study on chloroplast counting in Spruce needle leaves showed that examining the chemically fixed three-dimensional (3D) volume of dead mesophyll cells, using continuous optical cross-sections via confocal laser-scanning microscopy, provides more information than traditional two-dimensional (2D) images. The researchers found that nearly 90% of chloroplasts were missing in 2D images when dealing with thick cells (Kubínová et al., 2014). However, this method relies on complex data collection, introducing subjectivity and technical limitations. Some studies suggest that semi-automatic cell number quantification can be achieved using manual-thresholding segmentation and automated measurement with professional software like ImageJ. Nevertheless, overlapping cells may not be accurately distinguished and counted (Arena et al., 2017).

Deep learning has shown tremendous potential in plant research. It has been employed to accurately segment and analyze individual cells in microscopic images (van Valen et al., 2016; Xie et al, 2018). By merging transfer learning and Convolutional Neural Networks (CNNs), a deep-learning framework named DeeplearnMOR was developed for prompt classification of image categories and accurate identification of organelle abnormalities (Li et al., 2021). Additionally, an ImageJ plugin was created to enable non-experts to analyze data with U-Net for tasks like cell detection and shape measurements in biomedical images (Falk et al., 2019). Pachitariu et al. introduced Cellpose, a deep learning tool for precise cell segmentation across diverse image types. Although it does not directly count chloroplasts, Cellpose provides a foundation for analyzing chloroplasts within single cells (Pachitariu and Stringer, 2022). Recently, Lambret Frotte et al. developed Chloro-Count based on Mask Region-based CNNs, to count the chloroplast number in fixed rice bundle sheath cells using confocal laser scanning microscopy (Lambret Frotte et al., 2025). Meanwhile, the deep learning framework YOLO (You Only Look Once) object detection algorithm has transformed computer vision with its capacity to make real-time predictions at impressive speeds. It has been effectively used for cell detection and counting (Redmon and Farhadi, 2017; Aldughayfiq et al., 2023). Despite these advancements, it has not yet been applied to quantify subcellular structures like chloroplasts or mitochondria using 3D images from living cells.

To bridge the gap, we have developed DeepD&Cchl (Deep-learning-based Detecting-and-Counting-chloroplasts), a tool built on the YOLO object detection algorithm. The DeepD&Cchl, integrated with the Intersection Over Union (IOU) mode, enables accurate chloroplast counting in microscope images. When combined with the Cellpose segmentation tool, it allows for counting within individual 3D living cells. By plotting single cell chloroplast number against cell size, this method reveals a cell-type clustering effect, providing valuable morphological information for single-cell studies in plant research.

## 2 Materials and methods

### 2.1 Plant sample preparation

The original bryophyte plant materials, including *Sphagnum squarrosum* (*S. squ*)*, Physcomitrium patens* (*P. Pat*)*, and Ricciocarpos natans* (*R. nat*), were gifted by Prof. Ruiliang Zhu and Prof. Yue Sun from the School of Life Sciences at East China Normal University. The original *Wolffia australiana* (*W. Aus*) was gifted by Dr. Li Feng from the High School Affiliated to Renmin University of China.

For bryophyte culture, within a clean workbench, the plant’s surface soil was first rinsed off with clear water, followed by immersion in a 0.05% Triton buffer solution for 5 minutes. Subsequently, the specimen was treated with a 5% NaClO solution for another 5 minutes and then rinsed with sterilized distilled water three times, each rinse lasting 2 minutes. Excess moisture on the surface of the thallus was absorbed using sterile filter paper. The specimen was then placed on a pre-prepared ½ GB5 medium with 1% sucrose, sealed with a sealing film, and then incubated in a room maintained at a constant temperature of 22°C with a light cycle of 16 hours (at 800 lux) to 8 hours.

For cultivation, thallus sections that were vibrant green and in good growth condition were placed on the same medium for propagation. During this process, the growth status of the scales was observed and documented. The culture medium was prepared by dissolving Gamborg B5 Medium powder and sucrose in distilled water, adjusting the pH to 5.7-5.8, adding agar, and then autoclaving. After sterilization, the medium was poured into Petri dishes and allowed to air-dry at room temperature.

For *Arabidopsis* Columbia (Col-0, WT) culture, the seedlings were transferred to soil 14 days after being cultured on 1/2 Murashige and Skoog (MS) agar medium in a controlled growth chamber (16-h-light/8-h-dark photoperiod, 22°C and 70% relative humidity).

For *W. Aus* culture, the plants were cultured in the liquid 1/2 Murashige and Skoog (MS) medium in a controlled growth chamber.

### 2.2 Image collection

In this study, light microscope images of the samples were captured using an Olympus-BX43 biological microscope. Initially, we ensured the microscope slide was clean before adding 3–5 drops of distilled water onto it. Carefully, the liverwort scales were dissected using a dissecting needle and then laid flat in the water droplets on the slide. Once covered with a cover slip, we began our examination under the microscope at a low magnification to initially locate our target. Following this, we switched to a 10-fold eyepiece and a 60-fold objective magnifier for more detailed observation. After confirming regions where the chloroplasts were relatively dispersed without excessive clustering, photographs were taken, thus yielding a high-quality dataset of chloroplast images, setting the foundation for subsequent analysis. By carefully adjusting the longitudinal direction of the sample stage to move the microscope stage, a series of microscopic images (about 5–7 images) focusing on different planes were obtained. The image should include complete cells, ensuring that all chloroplasts in the cell could be clearly visible and counted.

The fluorescence microscope images were captured with a LEICA TCS SP8 (Germany) confocal microscope using a 40× objective or 63×objective oil immersion objective (excitation wavelengths: 632 nm).

For serial block face scanning electron microscopy (SBF-SEM) imaging, referring to a published protocol (Liu et al., 2024), the capitulum of *S. Squ* was fixed in a solution of 4% (w/v) paraformaldehyde in PBS at 4°C overnight. The fixed samples were stained with 2% (w/v) osmium tetroxide solution, treated with 1% (w/v) thiocarbohydrazide (TCH) solution, and restained with 1% aqueous uranyl acetate/Walton’s lead aspartate solution in turn. Samples were then washed and dehydrated through an ice-cold acetone series (25%, 50%, 75%, 100%, 100%, and 100% acetone) before being embedded in EPIN812 resin at room temperature. Blocks were modified into thin strips, mounted onto a stereomicroscope holder and then imaged and recorded using SEM (3VIEW-SEM, Zeiss). To obtain a three-dimensional image of the sample, a series of images (40 nm per slice) were obtained by removing each slice and imaging the next surface.

### 2.3 Dataset preparation

In this study, we captured approximately 300 bright-field light microscope images (191 images of *R. nat*, 51 images of *P. pat*, and 89 images of *S. Squ*), 119 fluorescence microscope images (*Wolffia Arrhiza*) and 512 SBF-SEM images (720×1200 pixels, *S. Squ*) for manual labeling.

After selecting images that excelled in terms of resolution, contrast, and chloroplast distribution, we employed an annotation tool, LabelImg software (https://github.com/tzutalin/labelImg; Tzutalin, 2015), to manually label the chloroplasts present in these images. Utilizing the bounding box feature of this tool, we meticulously assigned a label to each visible chloroplast. Over 20,000 chloroplasts for light microscopic images, 3600 chloroplasts for fluorescence microscope images and 2500 chloroplasts for the SBF-SEM images were labeled. A single annotator labeled the chloroplasts to eliminate inter-annotator variability to minimize potential impacts on model performance. 90% of the labeled chloroplasts were used as training data and the remaining 10% served as validation data. The annotated data were saved in.txt format, providing precise training and validation datasets for subsequent deep learning training.

### 2.4 Framework of DeepD&Cchl

**Figure 1:**
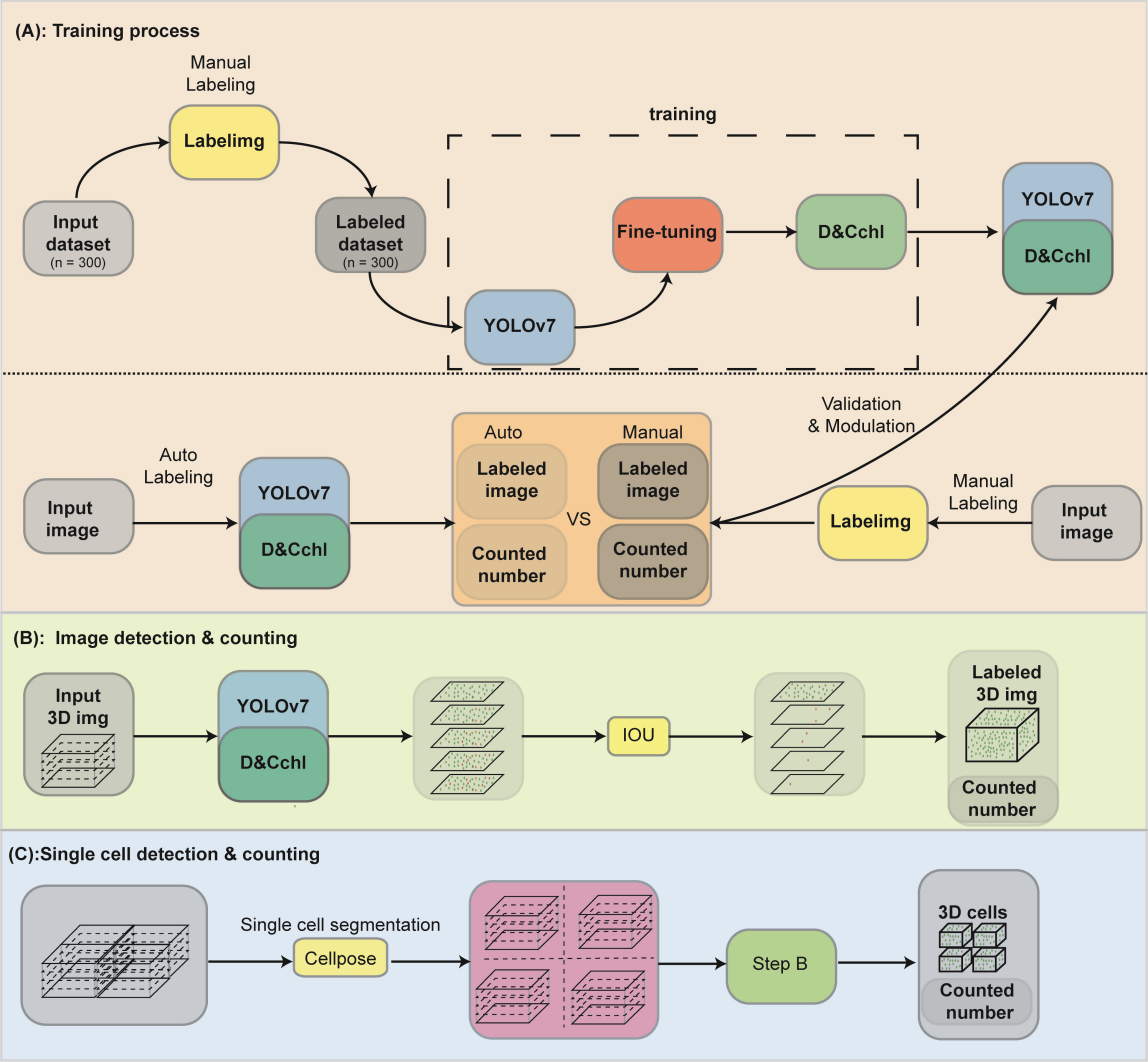
Workflow of the YOLOv7-DeepD&Cchl for chloroplast detection and counting. **(A)** For chloroplast detection with YOLOv7, microscopic plant cell images were collected and labeled using labelImg. The data was split into training and validation sets for DeepD&Cchl model training. Manually annotated data was used for comparison to train the DeepD&Cchl model. **(B)** Strategy and procedure for chloroplast detection and counting in 3D model generated from muti-layer stack. Five images from different focal planes were selected as input, and the Intersection over Union (IOU) strategy was integrated to avoid repeated detection. **(C)** The process for counting chloroplasts in individual cells involved the use of the Cellpose model. First, multi-layer images were input into the model for cell segmentation. These segmented images were then processed through the trained DeepD&Cchl model to accomplish chloroplast detection. Blocks colored in gray represented the dataset, yellow represented software, blue represented the neural network framework, and dark green represented the models generated from the training process.

The framework of DeepD&Cchl can be seen in **Figure 1**. DeepD&Cchl, a set of models based on YOLO (**Supplementary Figure S1**), were developed to detect and count chloroplasts. To maintain clarity and avoid confusion among DeepD&Cchl models, we established a systematic naming protocol: DeepD&Cchl_L for light microscope images, DeepD&Cchl_E for SBF-SEM images, and DeepD&Cchl_F for fluorescence microscope images. Each name reflects the unique features and applications of the corresponding model. The manually labeled datasets were used to train and tune the corresponding model (**Figure 1A**). For instance, light microscope chloroplast images of three bryophyte (*S. squ*, *P. pat*, and *R. nat*) were obtained (**Supplementary Figure S2**) for training of DeepD&Cchl-L model, which specifically detect light microscope chloroplasts. The well-manual-labelled light microscopy chloroplast dataset was fed to the YOLOv7 framework loaded with YOLOv7.pt model (https://github.com/WongKinYiu/yolov7; Wang et al., 2023).

To run YOLOv7 locally, we established Python 3.9 environment using “conda create -n yolov7_env python=3.9”, and then activate it with “conda activate yolov7_env”. Within this environment, we installed the necessary libraries and dependencies using “pip install -r requirements.txt” (https://github.com/WongKinYiu/yolov7), including PyTorch, torchvision, numpy, scipy, pandas, matplotlib, seaborn, and OpenCV. The YOLOv7 source code was also cloned from the same GitHub repository, and pretrained weights were downloaded for initialization. Details can be also referred to the attached Manual.

We trained each model for 500 epochs using the Adam optimizer with a learning rate of 0.001 and a batch size of 16 for each type of images. The training process lasted 2.662, 2.123 and 3.216 hours for DeepD&Cchl-L, DeepD&Cchl-F, and DeepD&Cchl-E, respectively. All of our training and testing data were stored in an input directory which contained separate folders for training and testing, along with the corresponding label txt files. Our experiments were conducted on a desktop computer equipped with an Intel Core i7–10700 CPU @ 3.80 GHz and an NVIDIA GeForce RTX 3060 with 12GB of VRAM. The code was executed using the PyTorch 2.0.0 framework and was supported by CUDA version 12.2.

To extend chloroplast detection to 3D cellular structures, we captured a sequential set of 2D images at different focal depths from the same sample. These consecutive 2D image layers were aligned according to their focal depths, sequentially stacked to form a 3D cell model, and processed by DeepD&Cchl to detect the chloroplasts in each layer. An IOU module was implemented to prevent repeated counting. The IOU was calculated as the ratio of the intersection to the union of chloroplast bounding boxes identified in two adjacent focal planes. Its values range from 0 (no overlap) to 1(complete overlap). Considering potential lateral displacement during focal adjustments, a threshold was chosen. The two chloroplasts with an IOU above the threshold were considered a single one. Otherwise, they were treated as distinct ones, incrementing the total count by one. This approach enabled accurate 3D volume quantification of chloroplasts (**Figure 1B**).

Additionally, for accurate counting of chloroplasts in individual cells, we employed the published Cellpose tool (https://www.github.com/mouseland/cellpose; Pachitariu and Stringer, 2022) to segment the images on a single-cell basis. Specifically, the pre-trained Cellpose model (cyto2) was fine tuned with plant cell images to create the suitable plant cell segmentation model Cyto2Pro, enabling a more efficient segmentation of single plant cells. Chloroplast counts were then calculated within each segmented cell (**Figure 1C**).

### 2.5 Model evaluation

We utilized manually labeled data as the ground truth to evaluate the performance of DeepD&Cchl. The mislabeled or omitted annotations were checked and counted manually. Several parameters were used to evaluate the performance, including precision (*P*), recall (*R*), average precision (*AP*), mean average precision (*mAP*) as well as F1 score (Rainio et al, 2024). Their definitions are shown in Equations 1–5.


(1) $$P=\frac{\text{TP}}{\text{TP}+\text{FP}}$$

(2) $$R=\frac{\text{TP}}{\text{TP+FN}}$$

(3) $$AP=\int_{0}^{1}P(R)\text{d}R$$

(4) $$mAP=\frac{1}{N}\sum_{i=1}^{N}AP_{i}$$

(5) $$\text{F}1=\frac{\mathrm{2TP}}{2TP+FP+FN}$$

In this context, True Positive (TP) represents the correctly detected number of target chloroplasts in the image, False Positive (FP) indicates the count of misidentifications, and False Negative (FN) signifies the number of particles in the image missed by the network. *P* is a metric measuring the ratio of correctly detected particles out of all detected chloroplasts, reflecting the model’s accuracy in object detection. On the other hand, *R* measures the ratio of correctly detected chloroplasts out of all chloroplasts in the sample, showcasing the model’s capability in detecting all target instances. *AP* is the mean precision value calculated across different recall levels, determined by the area under the precision-recall curve. *AP* offers insights into how well the model detects targets across various recall levels. Meanwhile, *mAP* is the average of *AP* values across all categories, providing an evaluation of the model’s overall performance. F1 score is the harmonic mean of precision and recall, used to comprehensively evaluate the balance between precision and recall of the model.

## 3 Results

Different DeepD&Cchl models, trained for different imaging modalities, were tested across diverse microscopy images. Their effectiveness in automated chloroplast counting was demonstrated in the following subsections through 2D, 3D, and single-cell analyses.

### 3.1 Accurate chloroplast counting in 2D light microscope images

**Figure 2:**
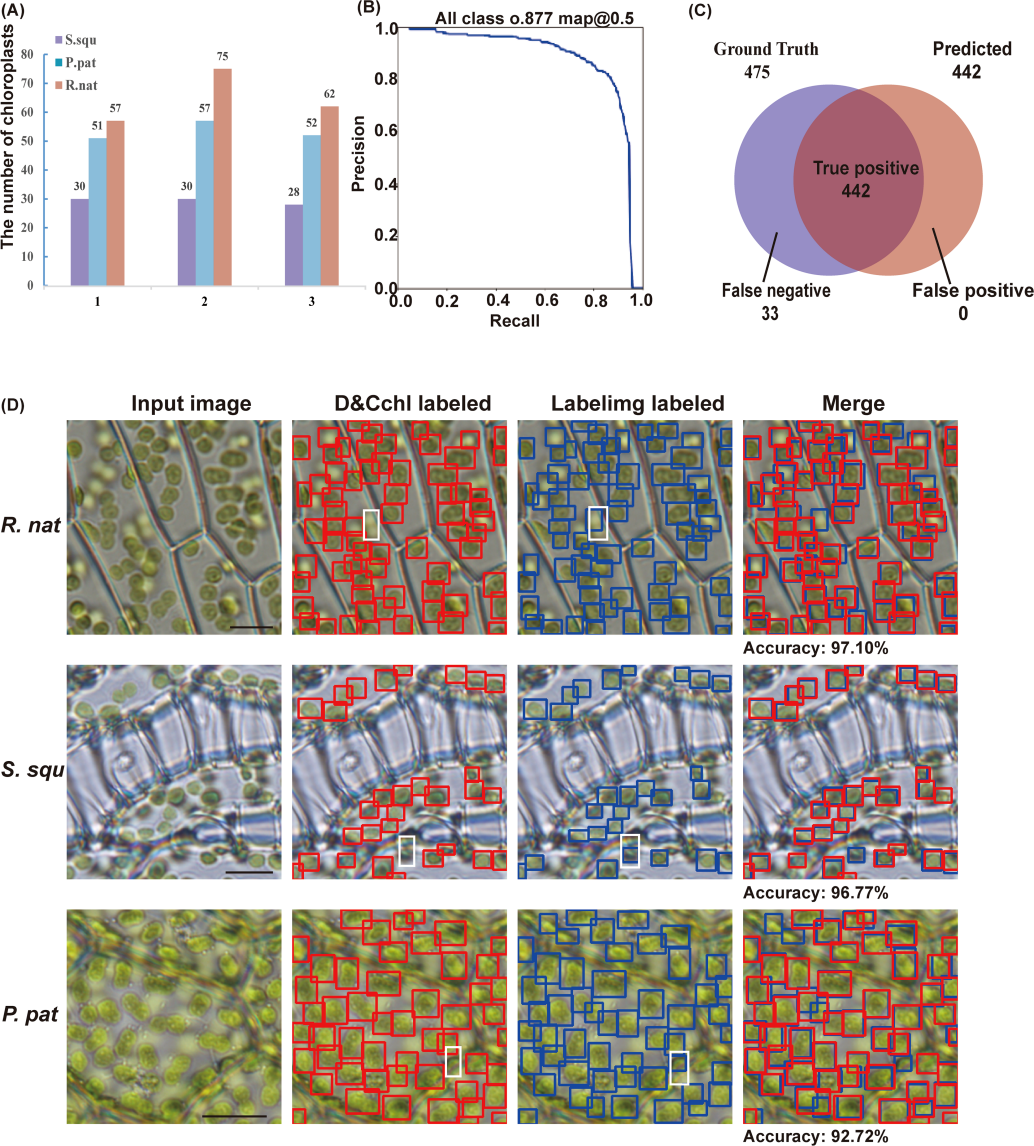
Assessment of the DeepD&Cchl. **(A)** The detection of chloroplasts from three images of *S. squ*, *P. pat*, and *R. nat*, respectively. **(B)** The precision-recall curve shows the model’s strong performance, achieving a precision of 87.7% at a 0.5 classification threshold. **(C)** Venn diagram showing the relationship between true positive set and predicted positive set, representing the DeepD&Cchl’s performance in chloroplast detection. **(D)** Input images were processed and chloroplasts were labeled with red boxes by DeepD&Cchl for comparison with chloroplasts that were manually labeled in blue boxes. Areas where the model failed to detect chloroplasts at different focal planes were highlighted by white dashed boxes.

To thoroughly assess the performance of DeepD&Cchl-L in chloroplast detection, we employed the comprehensive evaluation metric, *mAP*, which combines precision and recall (**Figure 2A**; **Supplementary Figures S3**, **S4**). In the validation dataset, when the confidence level was set to 0.5, the model achieved an average precision of 0.877. Furthermore, the F1 curve peaked at 0.84, further demonstrating the model’s excellent balance between precision and recall (**Supplementary Figure S5**).

To evaluate the efficiency of DeepD&Cchl in detecting chloroplast (**Figures 1B**, **2B**; **Supplementary Figure S6**), we manually labeled 9 unseen images (3 images from each different plant) as a test dataset. The DeepD&Cchl tool successfully detected 88 chloroplasts in *S. squ* (missing 3), 160 chloroplasts in *P. pat* (missing 10), 194 chloroplasts in *R.nat* (missing 20), and 475 chloroplasts in total images (missing 33), while falsely detected 0 chloroplasts (**Figure 2C**). The final counting results were calculated and the precision rates are 97.10%, 96.77%, and 92.72% respectively (**Figure 2D**). In all, the DeepD&Cchl-L tool showed an expert performance on automatic chloroplast detection and counting with light microscope images.

### 3.2 Counting in 3D volume with IOU module

**Figure 3:**
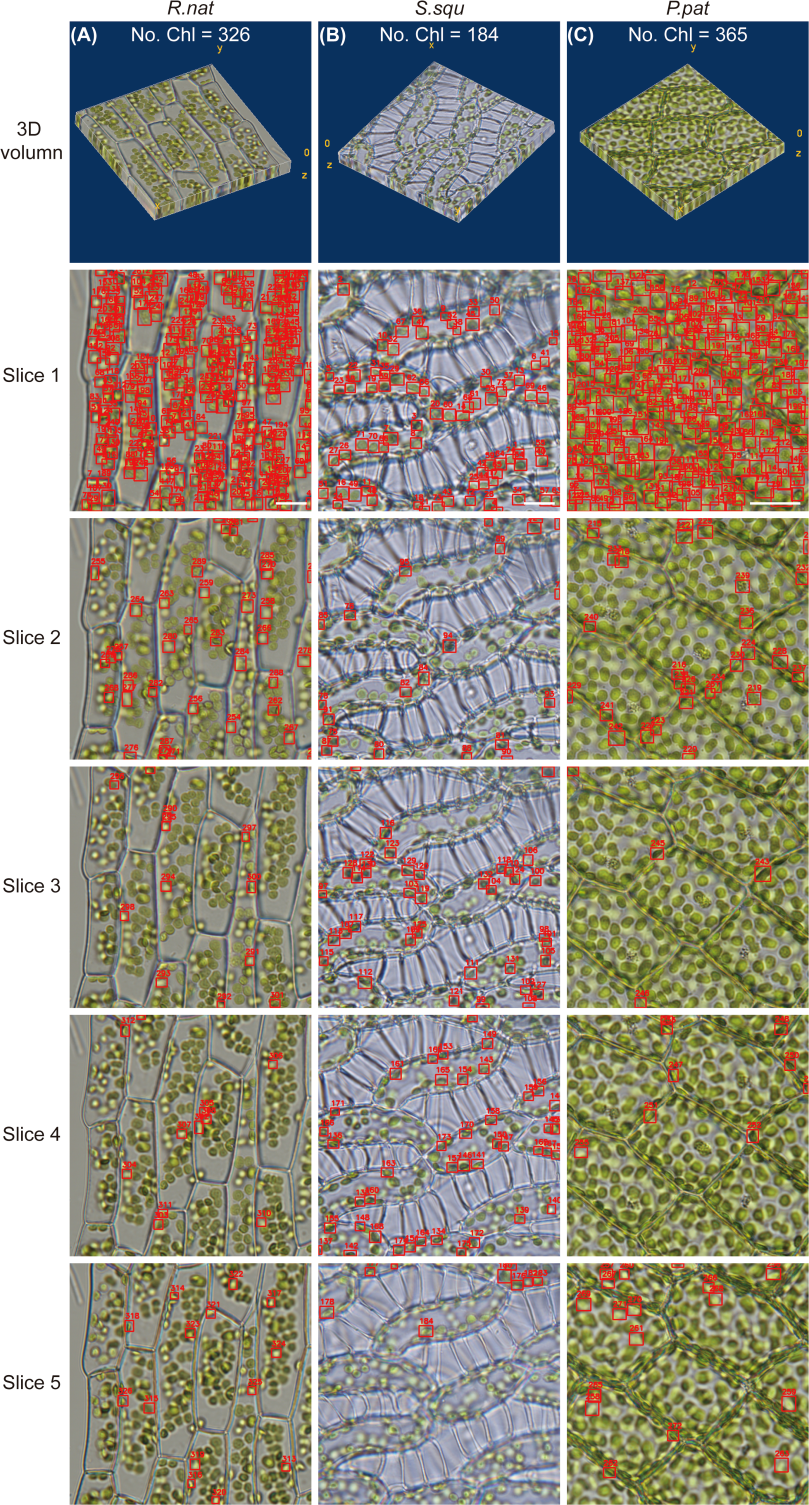
Chloroplast detection and counting using DeepD&Cchl in 3D volume. **(A-C)** The microscopic image series and chloroplast detection results for *S. squ*, *P. pat*, and *R. nat*, respectively. The labels Slice1 to 5 indicate the first to fifth layer of chosen images from each sample’s microscopic image series. All detected chloroplasts have been included in the respective benchmark. Scale bars, 10 μm.

To accurately calculate the chloroplast in 3D, we used DeepD&Cchl on a multilayer of light microscope images covering the entire cell (**Figures 1B** and **Figure 3**). To avoid duplicate counting, IOU calculations were performed between each detected target in the second image and the targets in the benchmark. The predetermined IOU threshold was set at 0.3. If the IOU exceeded the preset threshold, it was classified as an existing chloroplast in the benchmark to prevent duplicate counting; otherwise, it was identified as a new chloroplast and added to the benchmark as mentioned in Section 2.4 (**Figure 3**). Sequenced images of *S. squ*, *P. pat*, and *R.nat* leaf cells were obtained at various focal planes. The DeepD&Cchl was applied for each individual layer, and IOU was used to monitor the overlaps of every target between layers. The accurate counts of chloroplasts in different plants were obtained (**Figure 3**; **Supplementary Figure S7**). The precision was significantly improved in multilayer statistics than that in the single-layer image. For example, as shown in **Figure 3A**, about 254 chloroplasts were detected in the first layer, while the number was 326 in the total multilayer images. In **Figure 3B**, these numbers were 74 and 184, respectively, and in **Figure 3C**, they were 214 and 365. Overlaps between focused and out-of-focused chloroplasts might cause errors in detection and counting in a single layer. However, with the help of the IOU module, combined with training on focused chloroplast images, these errors were effectively minimized, yielding relatively accurate 3D quantification. These accurate 3D counts provide insights into chloroplast distribution, critical for investigating photosynthetic capacity.

### 3.3 Chloroplast detection for different imaging modalities

**Figure 4:**
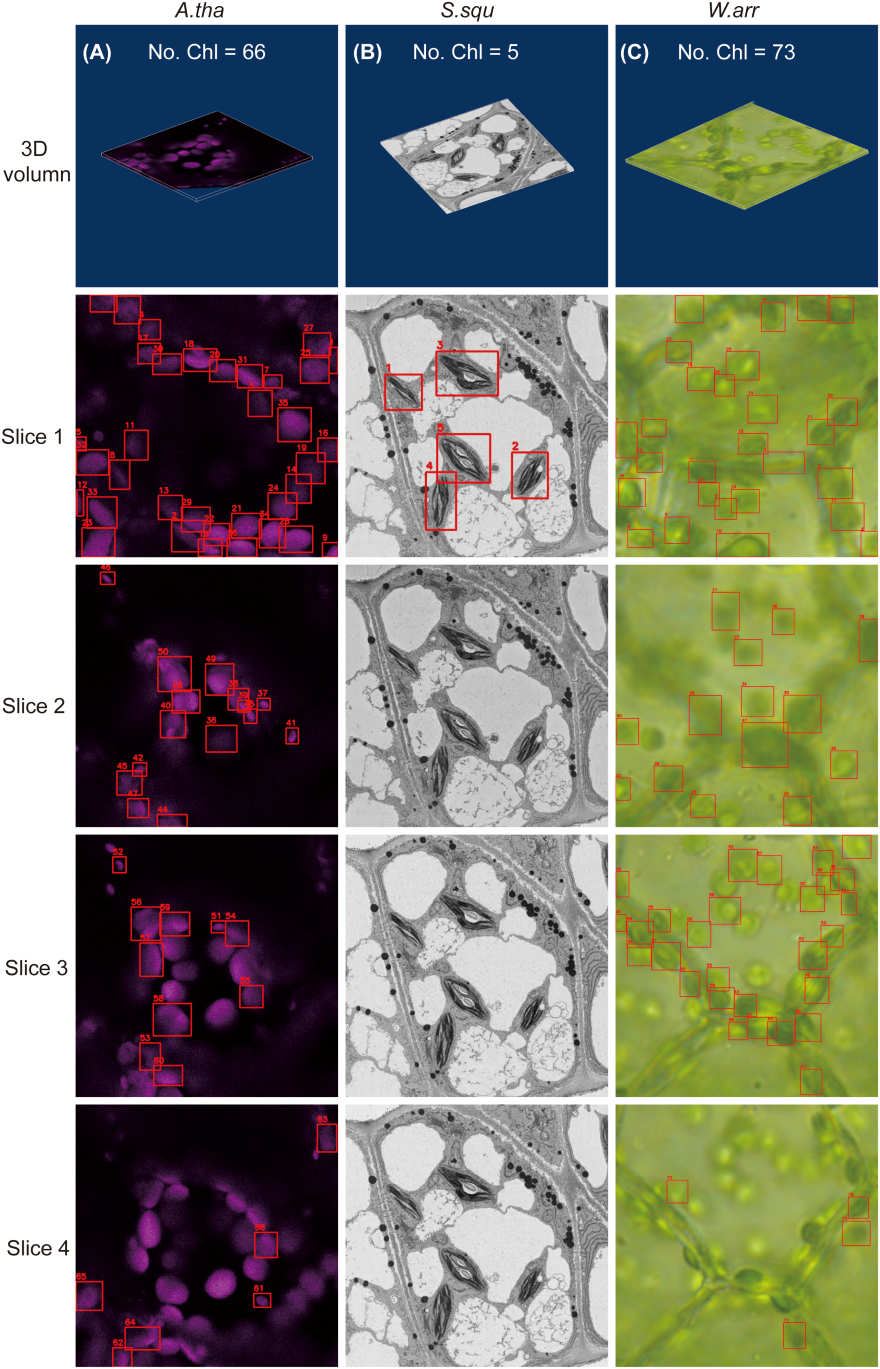
Applications of 3D volumetric detection using the DeepD&Cchl across different image types. **(A)** 3D chloroplast detection in fluorescent images of *A*. *tha*. **(B)** 3D chloroplast detection in electron microscopy images of *S. squ*. **(C)** 3D chloroplast detection in *W. aus*. The images were processed using the DeepD&Cchl_F **(A)**, DeepD&Cchl_E **(B)**, and DeepD&Cchl_L **(C)** models, respectively.

To expand the application of the DeepD&Cchl tool, we have incorporated various types of chloroplast microscope images, including SBF-SEM and fluorescence microscopy images (**Figure 4**). The same training strategies were used on various types of images, similar to those used for light microscope images. The evaluated metrics were also applied to DeepD&Cchl_E and DeepD&Cchl_F. In the corresponding validation dataset, at a confidence threshold of 0.5, DeepD&Cchl_E achieved an average precision of 0.927 and an F1 peak of 0.86 (**Supplementary Figures S8**-**S11**), while DeepD&Cchl_F attained a higher average precision of 0.973 and an F1 peak of 0.91 (**Supplementary Figures S12**-**S15**), both demonstrating excellent precision-recall balance.

To assess the performance of the three DeepD&Cchl models, we conducted tests on three distinct sets of untreated images (**Figure 4**; **Supplementary Figure S16**). The results revealed that 66, 5, and 73 chloroplasts were individually identified from fluorescence, SBF-SEM, and light microscope images, respectively. Notably, for the light microscope images, we purposely utilized a set from multilayer cell leaves of *W. Aus*. They exhibited inferior clarity compared to single-layered cell images (**Figure 4**). These results not only affirm the high adaptability and precision of the DeepD&Cchl tool in chloroplast detection and counting across various types and complexities of images, but also underscore the capability of deep learning methods in precise organelle quantification within diverse biological samples, highlighting their potential and universality in biological research. 

### 3.4 Single-cell chloroplast detection and cell type clustering

**Figure 5:**
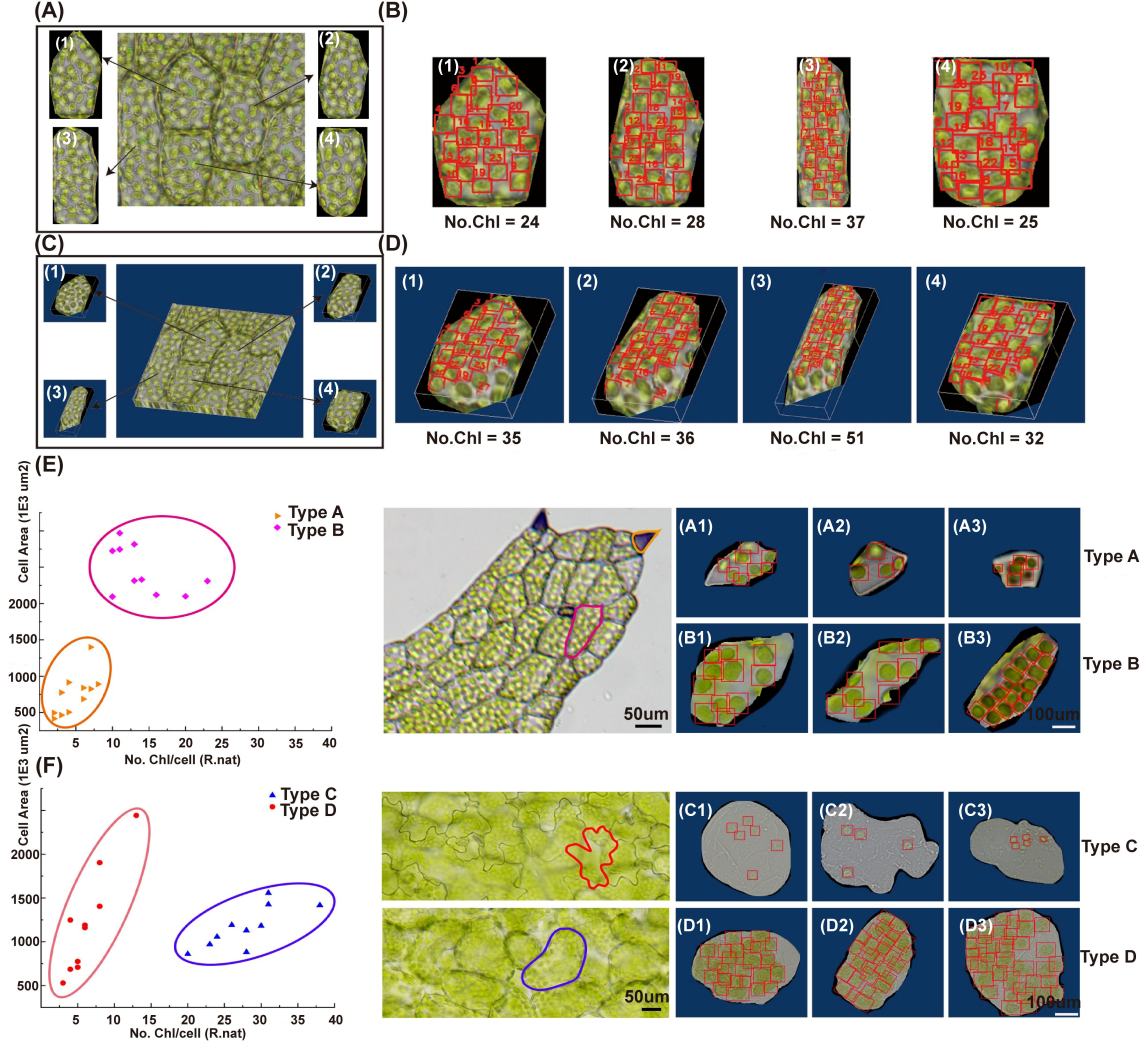
Single-cell chloroplast detection using DeepD&Cchl promotes cell type-specific clustering. **(A, B)** Single cell segmentation was performed via Cellpose for single-layer images, labeling cells 1–4 and detecting/counting Chloroplasts. **(C, D)** The same process was applied to multi-layer images, forming 3D volumes. For **(B, D**), “No.Chl” refers to the number of detected chloroplasts, highlighted by red boxes. **(E, F)** The scatter plots showed cell type-specific clustering by plotting chloroplast count against cell area in single cells of *R. nat* (, Type A-B) and (*A*) *tha* (Type C-D). The vertical axis signified cell size, the horizontal axis represented chloroplast count.

**Figure 6:**
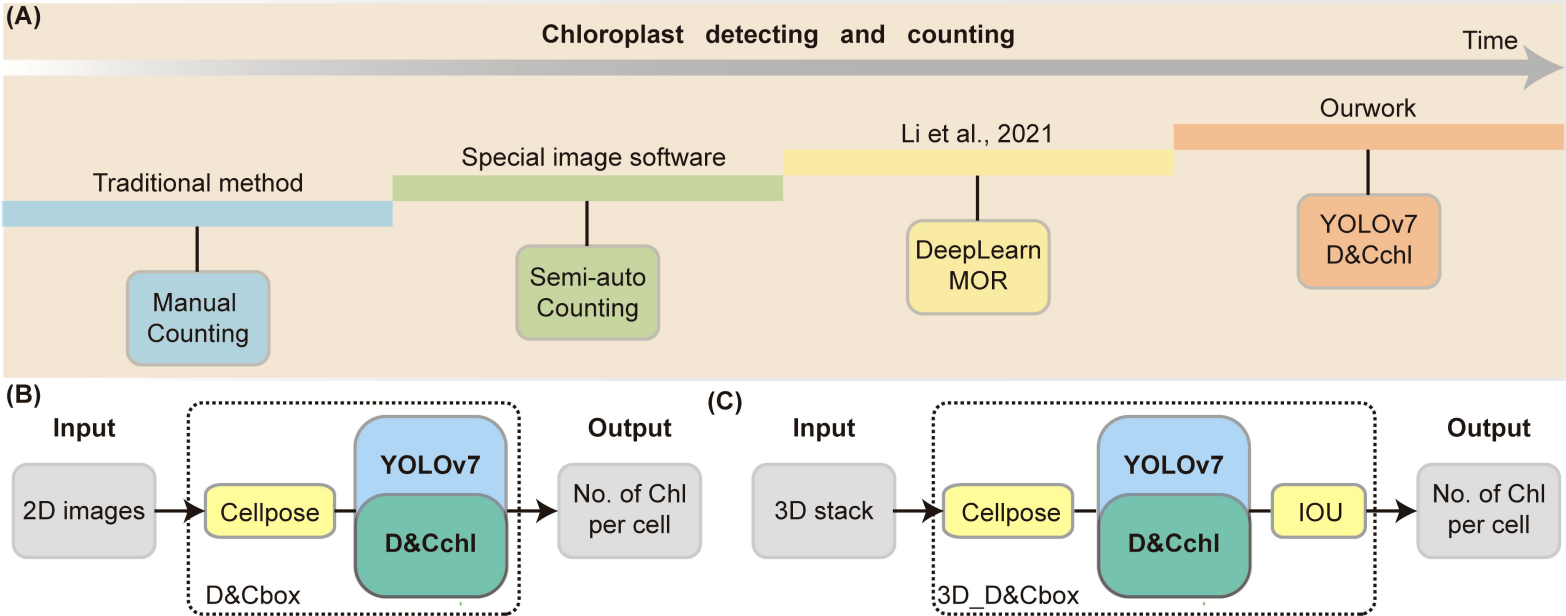
Summary of chloroplast detection techniques. **(A)** Overview of the major methods for counting chloroplasts, ranging from manual techniques to our proposed 3D single-cell detection method. **(B)** Process of chloroplast detection in 2D cells. **(C)** Workflow for chloroplast detection in 3D cells using series of multi-focal plane images.

To achieve single-cell counting of chloroplasts, we employed a Cellpose segmentation tool. We re-trained the original model cyto2 with our dataset and obtained a new model named ‘cyto2pro’ as mentioned in Section 2.4, and then utilized it to segment optical microscope images. We specifically chose intact individual cells as inputs for object detection, subsequently conducting chloroplast detection and counting within those individual cells using DeepD&Cchl at both 2D and 3D levels (**Figure 5**; **Supplementary Figures S17**, **S18**). Plotting cell area against chloroplast count in single cells of *R. nat* (Type A-B) and *Arabidopsis thaliana* (*A. tha*, Type C-D) reveals cell type-specific clustering (**Figure 6**). This highlights the relationship between chloroplast count and cell size in determining cell type, providing valuable morphological information for single-cell studies.

## 4 Discussions

### 4.1 The advancement of the DeepD&Cchl tool in plant cell biology

There are currently four main chloroplasts estimation methods widely used (**Figure 6**; **Table 1**): manual counting, semi-automated counting, deep-learning-based DeepLearnMOR (Li et al., 2021), and the DeepD&Cchl. Manual counting is simple but time-consuming, labor-intensive, and prone to errors. For instance, ImageJ offers a robust suite of counting tools like Cell Counter (Plugins-Analyze-Cell Counter) for manual labeling and auto-numbering (Arena et al., 2017). Semi-automated counting often needs user intervention and parameter setup for image segmentation and object counting. For example, ImageJ’s Analyze Particles feature allows one-click counting based on image pre-treatment with threshold-based segmentation (Rueden et al., 2017; Schneider et al., 2012). However, it’s worth noting that ImageJ has limitations in counting accuracy and lacks batch processing capabilities. Processing images one at a tim\

**Table 1:** A comprehensive comparison of chloroplast detection methods.

Method	Scope of observation	Staining (Yes/No)	Live Cell (Yes/No)	References
Manual	2D Slice	No	Yes	Lutz and Dzik, 1993
Semi-auto	2D Slice	No	Yes	Arena et al., 2017
DeepLearn MOR	2D Slice	Yes	No	Li et al., 2021
DeepD&Cchl	3D, whole cell	No	Yes	This study

can be time-consuming and inconvenient for large-scale data analysis. DeepLearnMOR, based on YOLOv5 (YOLO version 5), counts chloroplasts in fluorescence images. It offers high precision and speed in two-dimensional object detection and fully automated counting. However, the need for fluoresces images limits its practicality.

Our proposed tool, DeepD&Cchl, accurately counts chloroplasts in 3D single cells and is versatile across an unlimited number of plant species. As demonstrated in **Figures 6B, C**, either 2D or 3D images are subjected to cell segmentation using Cellpose and chloroplast auto-detection and counting using DeepD&Cchl. This method enables automated chloroplast detection and holds significant potential for widespread application in single-cell resolution correlation studies, linking single-cell transcriptomics with individual chloroplast status.

DeepD&Cchl introduces an innovative approach to plant cell research, particularly in understanding photosynthesis efficiency and plant adaptability to changing conditions. Usually, scientists perform chloroplast detection using single-layer images, because it’s challenging and time-consuming to avoid repeated chloroplast counting in multilayer images (Grishagin, 2015). In contrast, DeepD&Cchl offers an effective method to detect and count the chloroplasts in multilayer images. It addresses counting errors in 2D counting due to severe out-of-focus and defocusing issues, enhancing efficiency, particularly for studying organelles within whole cells. DeepD&Cchl enables precise quantification of chloroplasts, providing insights into a plant’s photosynthetic capacity, growth, and developmental stages (Xiong et al., 2017; Fujiwara et al., 2019). This tool can also monitor changes in chloroplast distribution in response to environmental stresses, aiding in the study of plant adaptability (Zahra et al., 2022; Liu et al., 2018; Feng et al., 2019).

### 4.2 Limitations and potential improvements for the DeepD&Cchl Tool

The current version of DeepD&Cchl includes three distinct chloroplast detection models trained for light microscope images, fluorescence microscope images, and SBF-SEM images, respectively. While effective within their specific scopes, these models require specialized knowledge for selection and operation, potentially limiting widespread adoption. To address this, further development of a multimodal model that can process diverse microscope images automatically is needed. Future research should focus on integrating various detection and classification techniques to create an efficient, accurate, and user-friendly chloroplast image analysis system. This will boost the model’s adaptability and flexibility, ensuring high-quality results across various observation scenarios.

The DeepD&Cchl method, utilizing deep learning, provides an innovative solution for accurately counting chloroplasts in plant cells. Its effectiveness would be amplified when integrated with object detection frameworks like Faster R-CNN (Faster Region-CNN) or SSD (Single Shot MultiBox Detector), improving precision and segmentation accuracy (Liu et al., 2016; Girshick et al., 2015). When combined with time series analysis tools like LSTM (Long Short-Term Memory), it can allow real-time monitoring of chloroplast dynamics under various environmental conditions (Hochreiter and Schmidhuber, 1997). The addition of multimodal data and technologies like autoencoders and VAEs (Variational Autoencoders) may enhance deep extraction of cellular features, offering a more efficient and comprehensive approach to plant cell research.

Despite these advances, limitations remain. For instance, the models may fail in out-of-focus cases or overlap errors in single-layer images (Section 3.2). Perhaps, some slightly out-of-focus images and overlapped cases in the training dataset could address these issues. However, beyond these limitations, DeepD&Cchl still has potential for broader applications within plant cells. Its robust and accurate image analysis makes it suitable for detecting and quantifying other cellular structures and organelles with further training. This could enrich our understanding of cellular mechanisms and processes, advancing plant cell biology.

## 5 Conclusion

In conclusion, DeepD&Cchl offers a reliable method for detecting and counting chloroplasts across various imaging modalities. Testing on light microscope images showed the precision rates of 92.72% to 97.10%, while multilayer analysis with the IOU module improved accuracy by reducing misses from out-of-focus and overlapping chloroplasts. It has been shown to accurately identify and count chloroplasts in fluorescence and SBF-SEM images. With Cellpose, it also enables chloroplast counting in single cells and cell classification based on these counts. These features provide insights into chloroplast distribution, potentially supporting the studies of photosynthetic capacity. However, limitations remain, including detection failures in out-of-focus cases and overlap errors in single-layer images. Additional training with diverse focus conditions may be needed to address the issues. Future efforts could refine 3D quantification techniques to enhance accuracy and accessibility for plant cell research.

## Data Availability

The data underlying this article are available in the article and in its online Supplementary Material. The raw dataset, as well as the scripts for the DeepD&Cchl model training and 17application macro, were shared on GitHub https://github.com/Shaokai9/AI4LifeScience_ECNU/tree/main/Deep%20subcellular%20detection.
