# Supplementary material for: DeepD&Cchl: an AI tool for automated 3D single-cell chloroplast detection, counting, and cell type clustering
Source: Front Plant Sci. 2025 May 23;16:1513953. doi: 10.3389/fpls.2025.1513953 (PMC12141212; doi:10.3389/fpls.2025.1513953)
Supplement: Supplementary file 1 [file DataSheet1.pdf]

# Step-by-Step Guide for Deep Learning Environment Setup and Image Analysis with the DeepD&Cchl tool

**This optimized guide provides step-by-step instructions for setting up the environment, running the code, and performing 2D/3D chloroplast detection and counting in single-cells. Feel free to let us know if you need further assistance!**

## Step 1. Set Up the Deep Learning Environment

*A deep learning environment including Pycharm, Anaconda, Pytorch, as well as CUDA and cuDNN needed to be set up. If you already have this environment set up or are familiar with the installation process, please skip to Step 2.*

To run the code, you need to configure a deep learning environment with PyCharm, Anaconda, PyTorch, CUDA, and cuDNN. Follow these steps:

### 1.1. Install Anaconda:

Download and install Anaconda from here (https://www.anaconda.com/download).

### 1.2. Create a Conda Environment:

Open Anaconda Prompt and create a new environment:

```
conda create -n ai4lifescience python=3.8
conda activate ai4lifescience
```

### 1.3. Install PyTorch with CUDA Support:

Install PyTorch and its dependencies. Replace cu11x with your CUDA version (e.g., cu118 for CUDA 11.8):

```
pip install torch torchvision torchaudio --index-url https://download.pytorch.org/whl/cu11x
```

### 1.4. Verify CUDA and cuDNN Installation:

Check if PyTorch recognizes your GPU:

```
import torch
print(torch.cuda.is_available())  # Should return True
print(torch.version.cuda)         # Should display your CUDA version
```

### 1.5. Install PyCharm:

Download and install PyCharm from here (https://www.jetbrains.com/pycharm/). Configure it to use the ai4lifescience Conda environment.

### 1.6 Check if the environment configuration is successful.

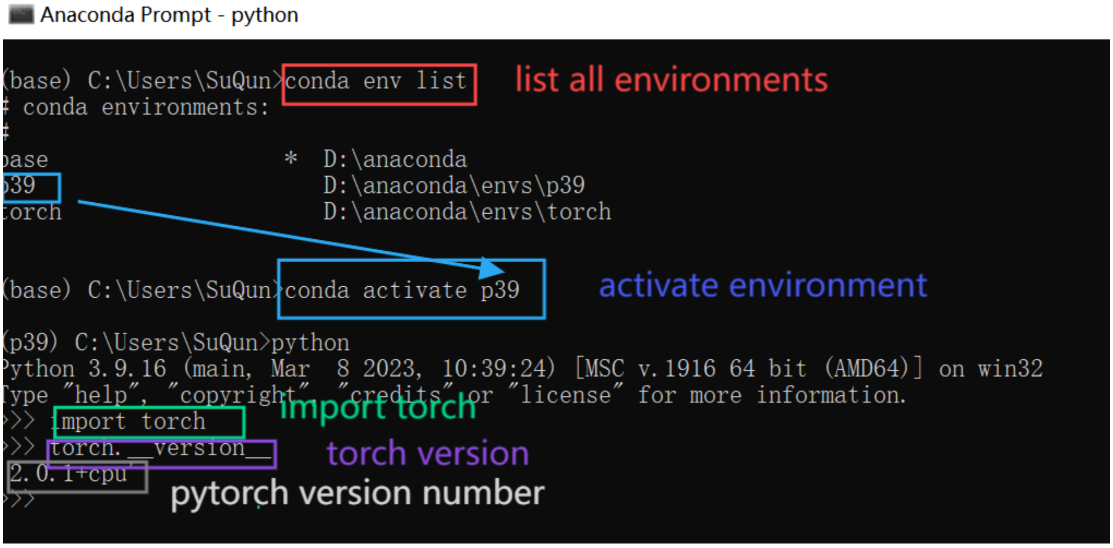


*The above steps can be also referred to "https://medium.com/@ragavan.arul26/how-to-run-the-yolov7-object-detection-model-on-windows-a4b9f8148ce3". For the Chinese version, please refer to the website here (https://blog.csdn.net/qq_67105081/article/details/139262362)*

## Step 2. Download the Code and Pretrained Models

### 2.1 Clone the GitHub Repository:

Download the source code from the GitHub repository:

```
git clone https://github.com/Shaokai9/AI4LifeScience_ECNU.git
cd AI4LifeScience_ECNU
```

Or go to GitHub to download the source code. (https://github.com/Shaokai9/AI4LifeScience_ECNU/tree/main)

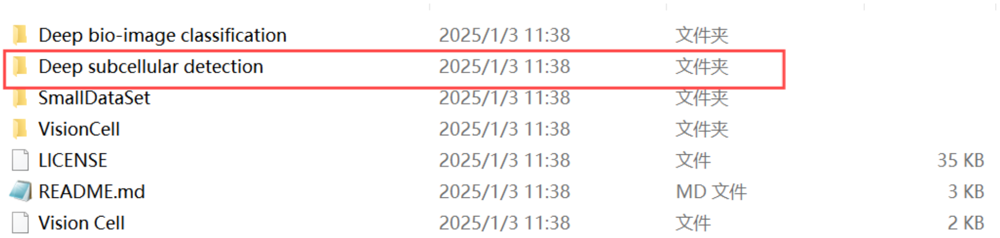


The above files are used for counting. The *requirements.txt* file is in the folder highlighted by the red square. Click on it to view its contents, as shown in the following figure.

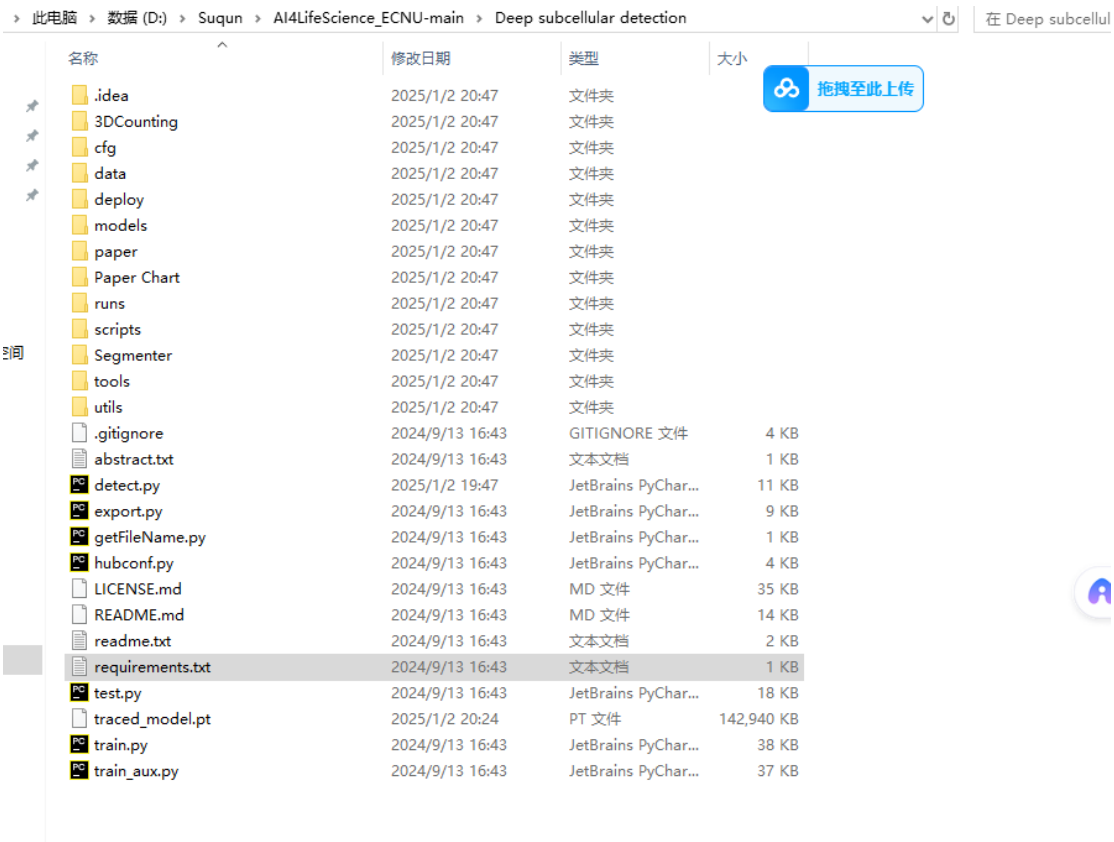


**2.2 Download Pre-trained Models and Test Images:**

Download the required files from Google Drive:

Pretrained models and test images: Google Drive Link (https://drive.google.com/drive/folders/1oCb9nsTbUv4KmX4bKbRin8CHFnGUs5nw?usp=sharing)

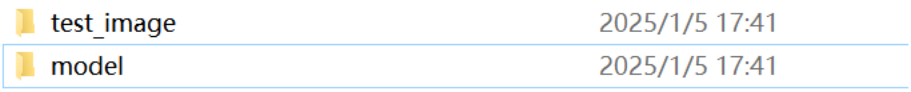


Place the downloaded files in your appropriate directories (e.g., models/ and data/).

# Step 3. Install Required Libraries

**3.1 Install Dependencies:**

Use the provided requirements.txt file to install the necessary libraries:

```
pip install -r requirements.txt
```

Enter the environment and install the required libraries based on the downloaded *requirement.txt* using pip. A screenshot is shown below: pip -r requirement.txt.

```
PS E:\AI4LifeScience_ECNU-main\Deep subcellular detection> pip install -r requirements.txt
Requirement already satisfied: matplotlib>=3.2.2 in d:\anaconda\lib\site-packages (from -r requirements.txt (line 4)) (3.9.1)
Requirement already satisfied: numpy<1.24.0,>=1.18.5 in d:\anaconda\lib\site-packages (from -r requirements.txt (line 5)) (1.23.5)
Requirement already satisfied: opencv-python>=4.1.1 in d:\anaconda\lib\site-packages (from -r requirements.txt (line 6)) (4.7.0.72)
Requirement already satisfied: Pillow>=7.1.2 in d:\anaconda\lib\site-packages (from -r requirements.txt (line 7)) (10.2.0)
Requirement already satisfied: PyYAML>=5.3.1 in d:\anaconda\lib\site-packages (from -r requirements.txt (line 8)) (6.0.1)
Requirement already satisfied: requests>=2.23.0 in d:\anaconda\lib\site-packages (from -r requirements.txt (line 9)) (2.32.2)
Requirement already satisfied: scipy>=1.4.1 in d:\anaconda\lib\site-packages (from -r requirements.txt (line 10)) (1.14.0)
Requirement already satisfied: torch!=1.12.0,>=1.7.0 in d:\anaconda\lib\site-packages (from -r requirements.txt (line 11)) (2.0.1)
Requirement already satisfied: torchvision!=0.13.0,>=0.8.1 in d:\anaconda\lib\site-packages (from -r requirements.txt (line 12)) (0.15.2
Requirement already satisfied: tqdm>=4.41.0 in d:\anaconda\lib\site-packages (from -r requirements.txt (line 13)) (4.66.4)
Requirement already satisfied: protobuf<4.21.3 in d:\anaconda\lib\site-packages (from -r requirements.txt (line 14)) (3.20.2)
Collecting tensorboard>=2.4.1
  Downloading tensorboard-2.18.0-py3-none-any.whl (5.5 MB)
     ━━━━━━━━━━━━━━━━━━━━━━━━━━━━━━━━━━━━━━━━ 5.5/5.5 MB 2.0 MB/s eta 0:00:00
```

### 3.2 Verify Installation:

Ensure all libraries are installed without errors.

## Step 4. Configure and Run 2D Object Detection

```python
if __name__ == '__main__':
    parser = argparse.ArgumentParser()
    parser.add_argument( *name_or_flags: '--weights', nargs='+', type=str, default=r'C:\Users\Administrator\Desktop\1/best.pt', help='model.pt path(s)')
    parser.add_argument( *name_or_flags: '--source', type=str, default=r'C:\Users\Administrator\Desktop\2', help='source')  # file/folder, 0 for webcam
    parser.add_argument( *name_or_flags: '--img-size', type=int, default=640, help='inference size (pixels)')
    parser.add_argument( *name_or_flags: '--conf-thres', type=float, default=0.4, help='object confidence threshold')
    parser.add_argument( *name_or_flags: '--iou-thres', type=float, default=0.4, help='IOU threshold for NMS')
    parser.add_argument( *name_or_flags: '--device', default='', help='cuda device, i.e. 0 or 0,1,2,3 or cpu')
    parser.add_argument( *name_or_flags: '--view-img', action='store_true', help='display results')
    parser.add_argument( *name_or_flags: '--save-txt', action='store_true', default=True,  help='save results to *.txt')
    parser.add_argument( *name_or_flags: '--save-conf', action='store_true', help='save confidences in --save-txt labels')
    parser.add_argument( *name_or_flags: '--nosave', action='store_true', help='do not save images/videos')
    parser.add_argument( *name_or_flags: '--classes', nargs='+', type=int, help='filter by class: --class 0, or --class 0 2 3')
    parser.add_argument( *name_or_flags: '--agnostic-nms', action='store_true', help='class-agnostic NMS')
    parser.add_argument( *name_or_flags: '--augment', action='store_true', help='augmented inference')
    parser.add_argument( *name_or_flags: '--update', action='store_true', help='update all models')
    parser.add_argument( *name_or_flags: '--project', default=r'C:\Users\Administrator\Desktop\3', help='save results to project/name')
    parser.add_argument( *name_or_flags: '--name', default='exp', help='save results to project/name')
    parser.add_argument( *name_or_flags: '--exist-ok', action='store_true', help='existing project/name ok, do not increment')
    parser.add_argument( *name_or_flags: '--no-trace', action='store_true', help='don`t trace model')
    opt = parser.parse_args()
    print(opt)
```

model.pt path

Test images path

save path

*The above modifications are made in the detect.py file.*

### 4.1 Modify the Weights Path:

Open the configuration file (e.g., config.yaml) and update the path to the pretrained model (best.pt).

```yaml
weights: C:\Users\Administrator\Desktop\1\best.pt
```

*Here, the local path is "C:\Users\Administrator\Desktop\1\best.pt". In acutural use, the users should change the path to their local path.*

### 4.2 Set the Test Data Path:

Update the path to the directory containing your test images:

```yaml
test_data: C:\Users\Administrator\Desktop\2
```

*In this fold, you can see the following two figures for testing.*

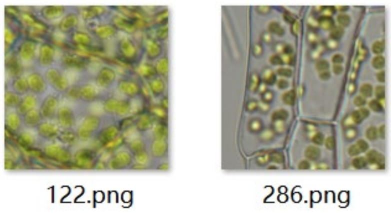


**4.3 Set the Output Path:**

Specify the directory to save the results:

```yaml
output: C:\Users\Administrator\Desktop\3
```

**4.4 Run the 2D Detection Script:**

Execute the script to perform 2D object detection:

```
PS E:\AI4LifeScience_ECNU-main\Deep subcellular detection> python detect.py
```

**4.5 Check Results:**

The output images with detected objects will be saved in the specified output directory.

*The following figures show the successful results for the tested cases.*

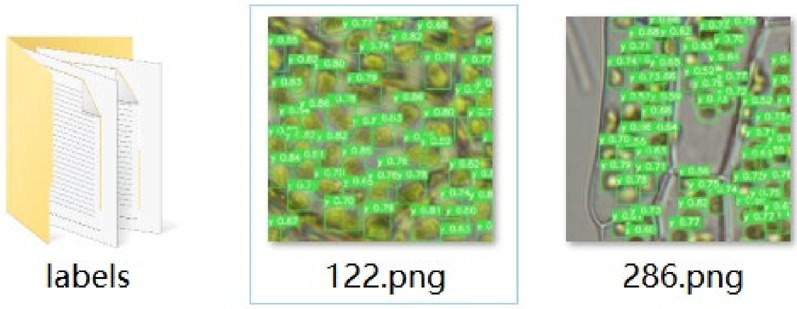

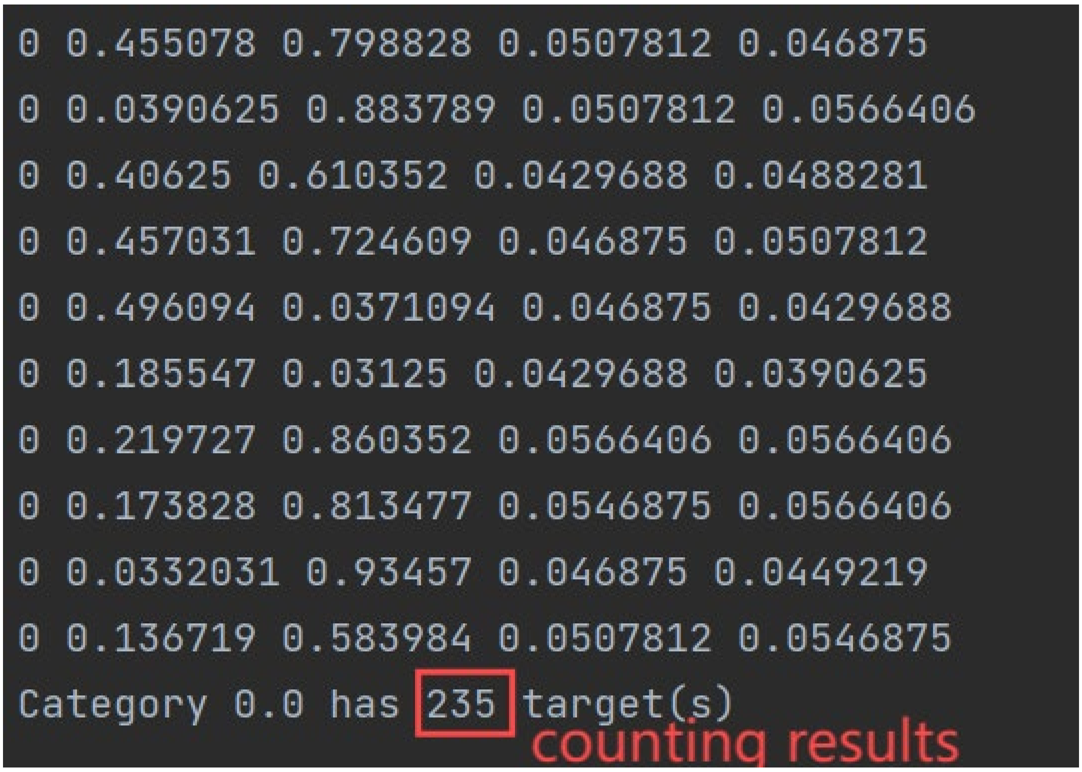


## <u>Step 5. Perform 3D Counting</u>

**5.1 Modify the 3D Counting Script:**

Open 3DCounting.py and update the paths for input images, and output directory similar as the above steps. A set of consecutive images and their corresponding 2D detection result text files (.txt) are required here.

*Here are the example input images and labels:*

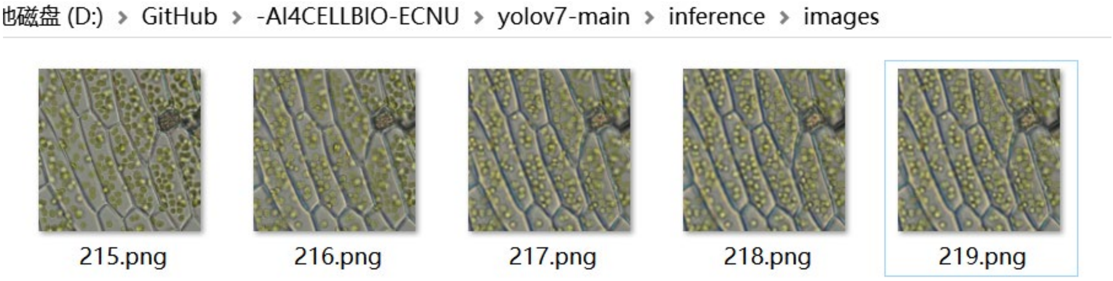


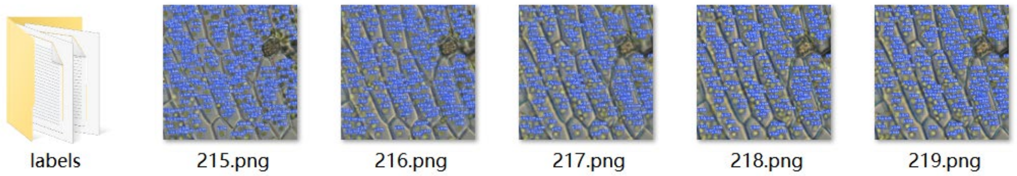


*The path for the output directory.*

```python
# Set your own paths here
txt_folder = r'C:\Users\Administrator\Desktop\3\exp2\labels'
output_txt_path = r'C:\Users\Administrator\Desktop\5'
img_folder = r'C:\Users\Administrator\Desktop\2'
output_img_folder = r'C:\Users\Administrator\Desktop\4'
```

**5.2 Run the 3D Counting Script:**

Execute the script to perform 3D counting:

```
python 3DCounting.py
```

The 3D counting results are shown below.

ID: 282, BBox (normalized): [0.484375, 0.143555, 0.0351562, 0.0449219], Source: 3.txt
ID: 283, BBox (normalized): [0.743164, 0.0283203, 0.0371094, 0.0371094], Source: 3.txt
ID: 284, BBox (normalized): [0.855469, 0.487305, 0.03125, 0.0332031], Source: 3.txt
ID: 285, BBox (normalized): [0.124023, 0.757812, 0.0449219, 0.0507812], Source: 4.txt
D: 286, BBox (normalized): [0.919922, 0.279297, 0.0429688, 0.0429688], Source: 5.txt

**5.3 View Results:**

The 3D counting result (e.g., 286) will be displayed in the terminal or saved in a log file.

## Step 6. DeepD&Cchl application in single cells

**6.1 Install Cellpose:**

Install Cellpose and its dependencies:

```
pip install cellpose
```

**6.2 Run Cellpose for Segmentation:**

Use Cellpose to segment single cells from 2D images:

```
python -m cellpose --dir C:\Users\Administrator\Desktop\2 --pretrained_model cyto --save_tif
```

**6.3 Process Segmentation Results:**

Use the segmenter.py script to process the .npy files generated by Cellpose:

```
python segmenter.py --input C:\Users\Administrator\Desktop\2 --output C:\Users\Administrator
\Desktop\3
```

**6.4 Analyze Single-Cell Chloroplast status**:

Follow **steps 4–5** to analyze the chloroplast status of single cells.

*******************************************************************************

## Troubleshooting and Tips

1. **CUDA Errors:** Ensure your GPU drivers, CUDA, and cuDNN versions are compatible with

PyTorch.

2. **Missing Libraries**: Double-check requirements.txt and install any missing packages manually.

3. **Path Issues**: Use absolute paths to avoid file-not-found errors.

4. **Detailed usage of Cellpose** (Additional details can also be found in this YouTube video: https://www.youtube.com/watch?v=5qANHWoubZU. In our work, we also referred to the Chinese tutorial on Zhihu: [https://zhuanlan.zhihu.com/p/652546486](https://zhuanlan.zhihu.com/p/652546486).)

4.1. Use Cellpose to segment 2D images and save the segmentation results in .npy files. This process is implemented according to segmenter.py."

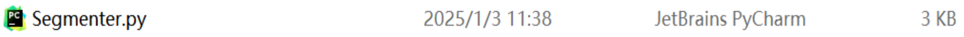


4.2. After obtaining a set of segmented single-cell images, configure the function parameters with your file paths. Follow **steps 4 to 5** to count cells in the entire image and obtain the single-cell result

```
def segment_images(image_folder, mask_folder, output_dir):
```
