# Supplementary material for: DeepD&Cchl: an AI tool for automated 3D single-cell chloroplast detection, counting, and cell type clustering
Source: Front Plant Sci. 2025 May 23;16:1513953. doi: 10.3389/fpls.2025.1513953 (PMC12141212; doi:10.3389/fpls.2025.1513953)
Supplement: Supplementary file 2 [file DataSheet2.pdf]

Supplemental Figure S1

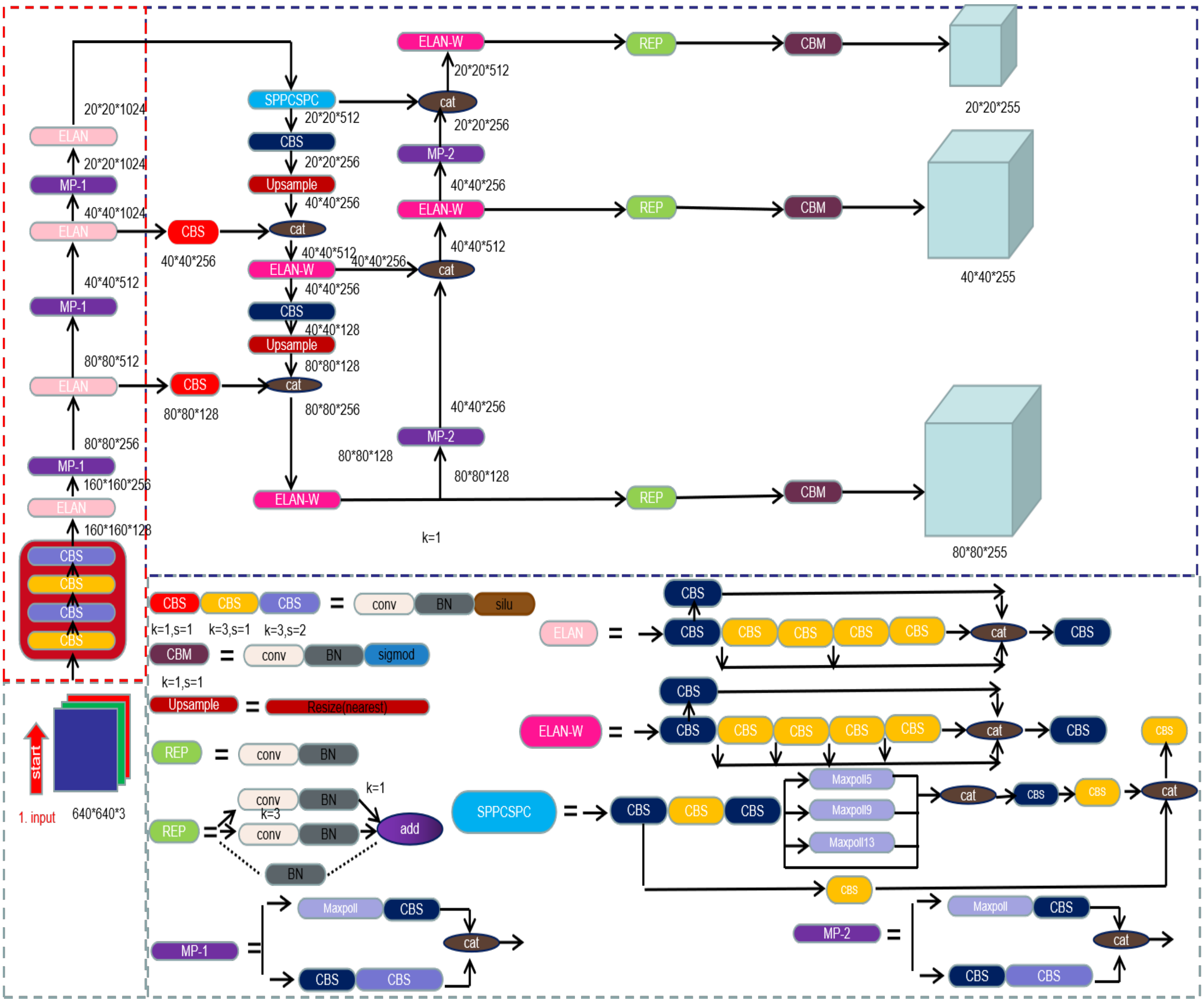

Supplemental Figure S1 Network structure diagram of Yolov7 algorithm

The YOLOv7 network structure consists of four parts: Firstly, the input section handles image preprocessing, such as data augmentation and size adjustment. Next, the backbone feature extraction network deeply extracts features from the image using CBS convolution, MP convolution, and ELAN layers. Subsequently, the neck feature fusion network integrates multi-scale features through CBS, SPPCSPC, MP, and ELAN structures. Finally, the detection head employs an anchor mechanism for object detection, utilizing CIOU and NMS processing to achieve precise prediction results.

Supplemental Figure S2

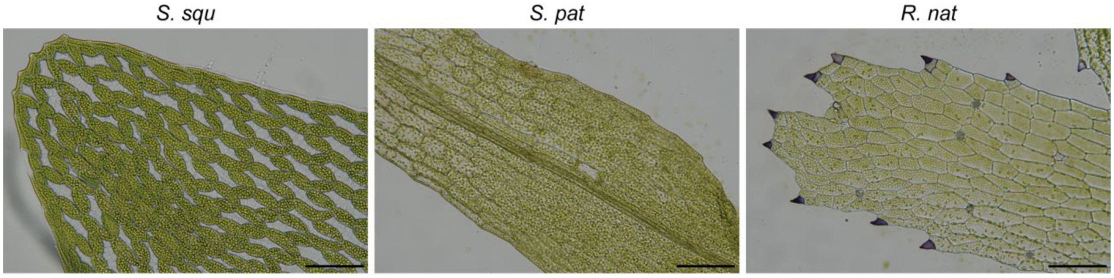

Supplemental Figure S2 The leaves of the three plant materials were used for the establishment of the DeepD&Cchl tool

The cells in the plant leaves from these bryophytes exhibited a regular distribution and shape. *S. squ* is *Sphagnum squarrosum*, *P. pat* is *Physcomitrium patens*, and *R. nat* is *Ricciocarpos natans*.

Supplemental Figure S3

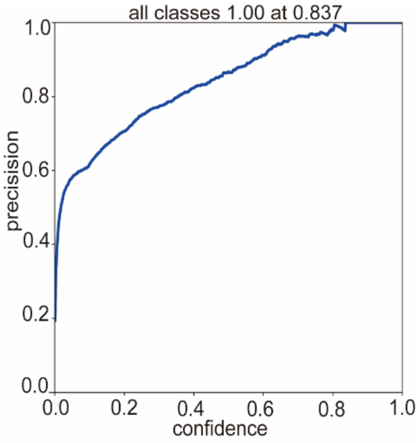

Supplemental Figure S3 The Precision Curve of DeepD&Cchl_L

Provides insights into the model's positive predictive accuracy at different confidence thresholds. Notably, the model boasts a 100% precision rate at an 83.7% confidence threshold.

Supplemental Figure S4

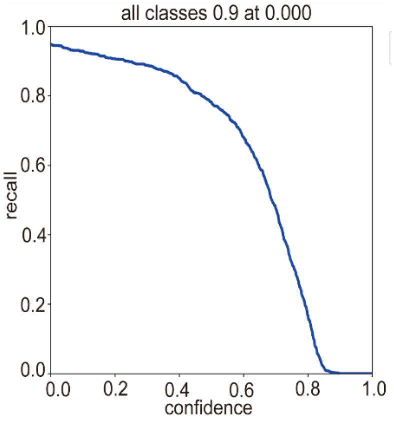

Supplemental Figure S4 The Recall Curve of DeepD&Cchl_L

Sheds light on the model's capability to correctly recognize positive instances across varying confidence thresholds. At its peak, the recall value stands at 90%, underlining the model's adeptness in identifying genuine chloroplasts.

Supplemental Figure S5

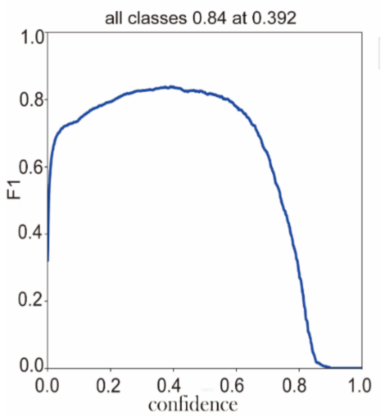

Supplemental Figure S5 The F1 Score Curve of DeepD&Cchl_L

Illustrates the model's F1 score performance, representing a balance between precision and recall. At a 39.2% confidence threshold, the F1 score peaks at 84%.

Supplemental Figure S6

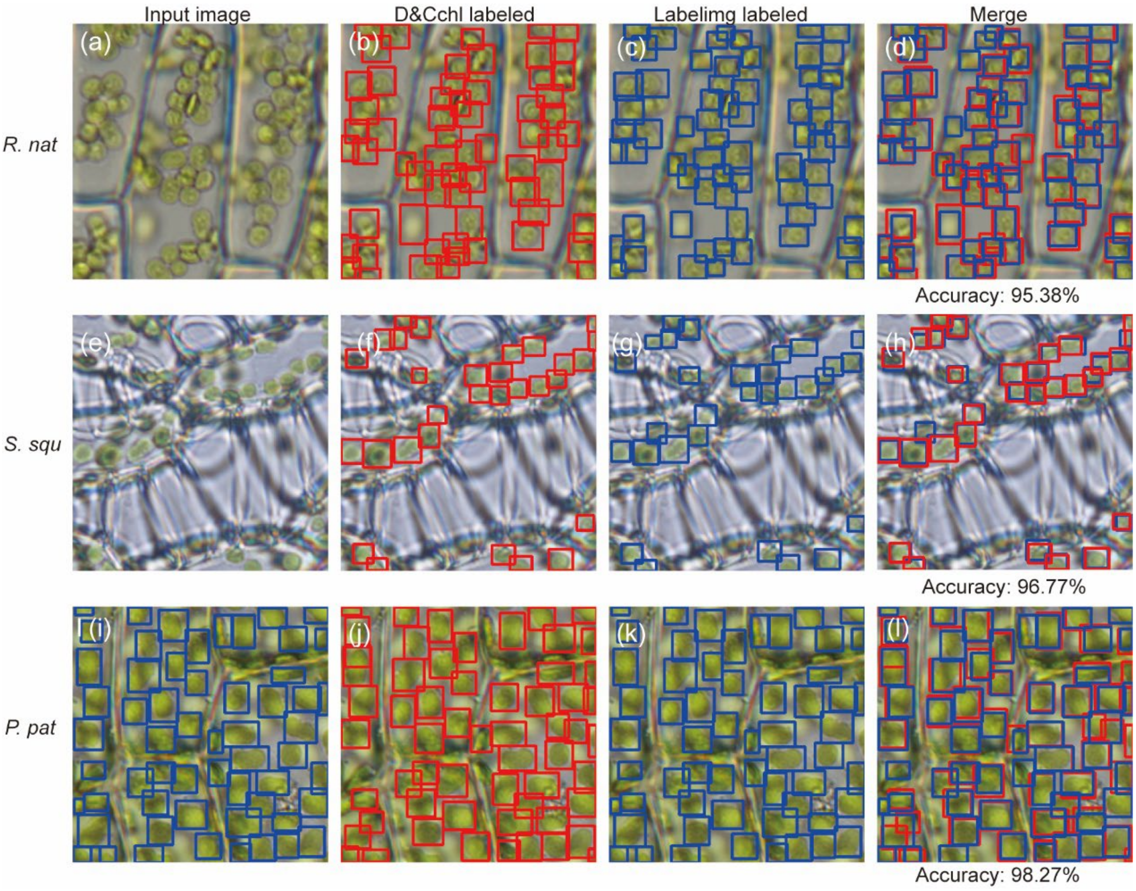

Supplemental Figure S6 Detection of chloroplasts using DeepD&Cchl_L in 2D plant cells

Detailed detection and evaluation of chloroplasts in individual microscopic plant cell images. (a, e, i) Original microscopic images of the cells from *Ricciocarpos natans*, *Sphagnum squarrosum*, and *Physcomitrium patens*, respectively, showcasing the intrinsic distribution of chloroplasts. (b, f, j) Images processed by the D&Cch, indicating the chloroplasts detected automatically. (c, g, k) Manual annotations on the images marking each chloroplast, serving as the ground truth for evaluation. (d, h, l) Combination of automatic detection results and manual annotations, highlighting the overlap and differences. The detection accuracy for the three plant cells was 93.75%, 96.55%, and 91.22%, respectively.

Supplemental Figure S7

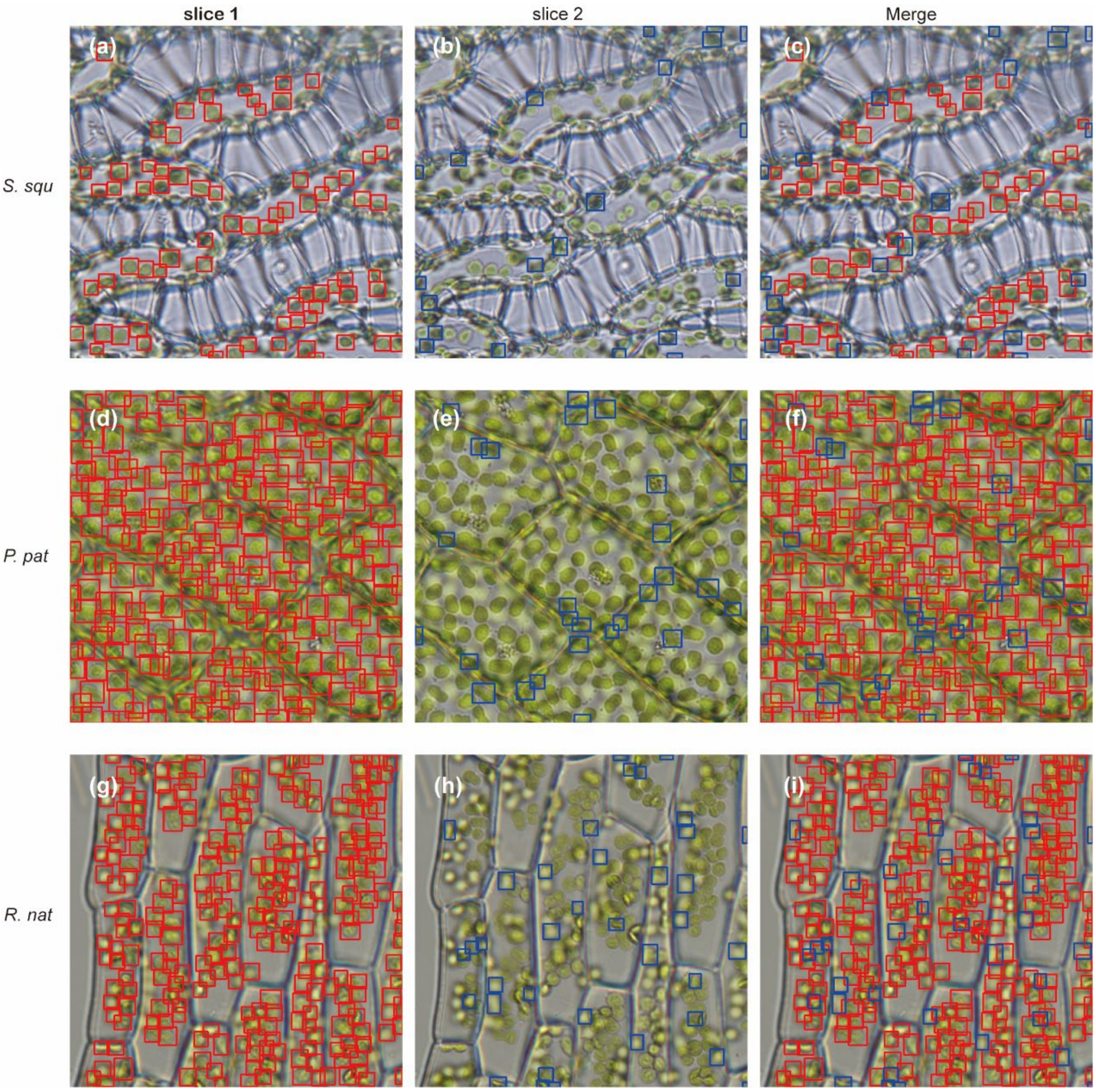

Supplemental Figure S7 Detection of chloroplasts using DeepD&Cchl in 3D plant cells

Related with Figure 3, "Slice1" was selected as the initial image in each series, "Slice2" represents the second image in each series.We conducted IOU calculations for each detected target in the second image against targets in the benchmark, eliminating counts that overlap with "Slice1".

Supplemental Figure S8

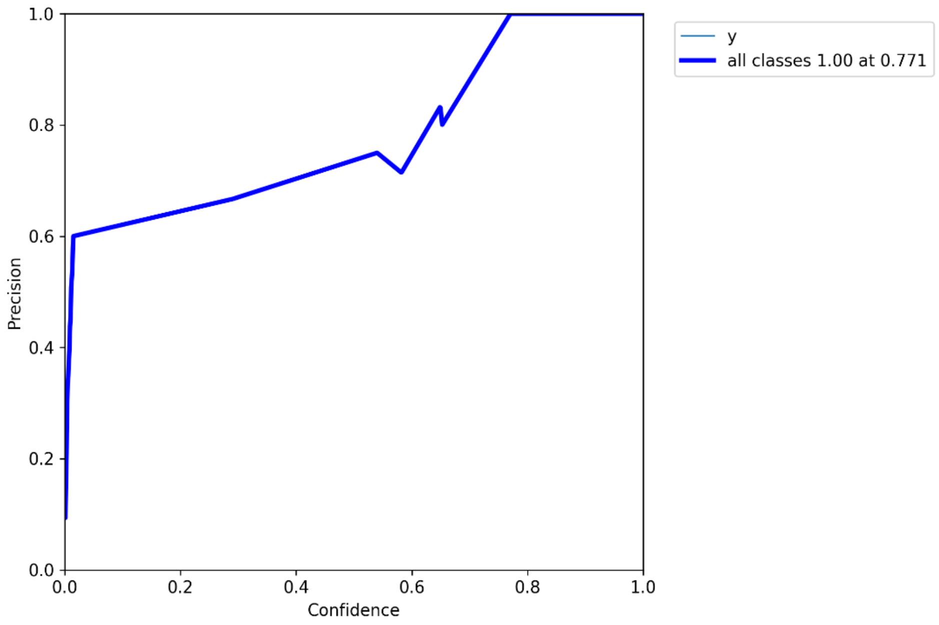

Supplemental Figure S8 The Precision Curve of DeepD&Cchl _E

Supplemental Figure S9

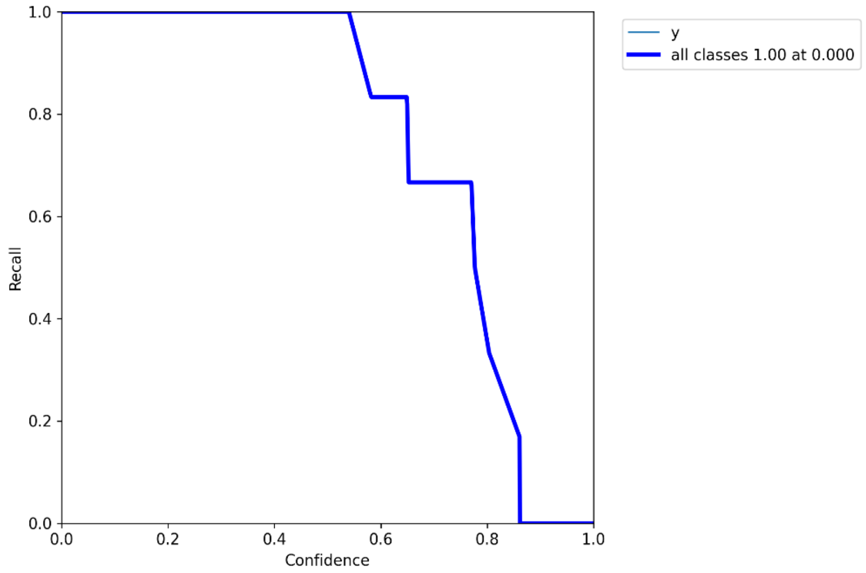

Supplemental Figure S9 The Recall Curve of DeepD&Cchl _E

Supplemental Figure S10

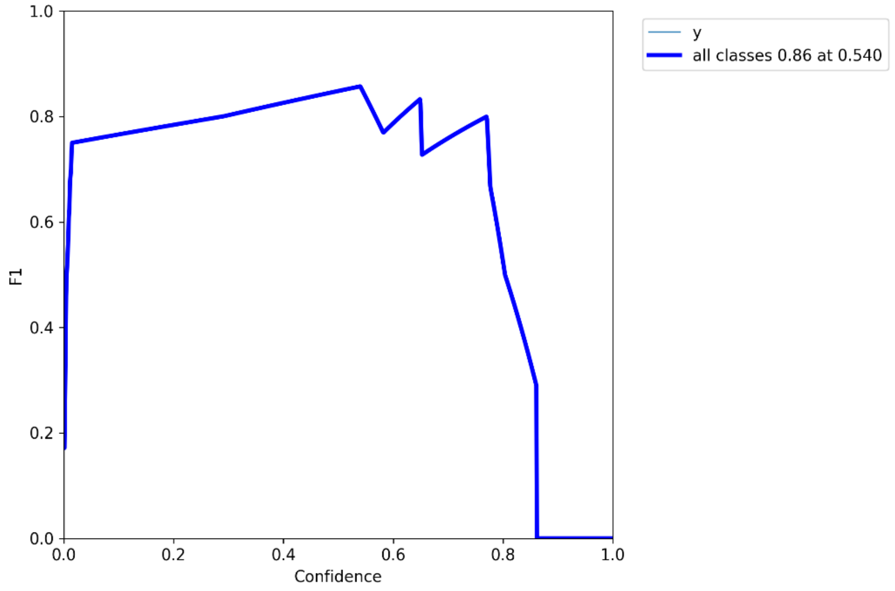

Supplemental Figure S10 The F1 Score Curve of DeepD&Cchl _E

Supplemental Figure S11

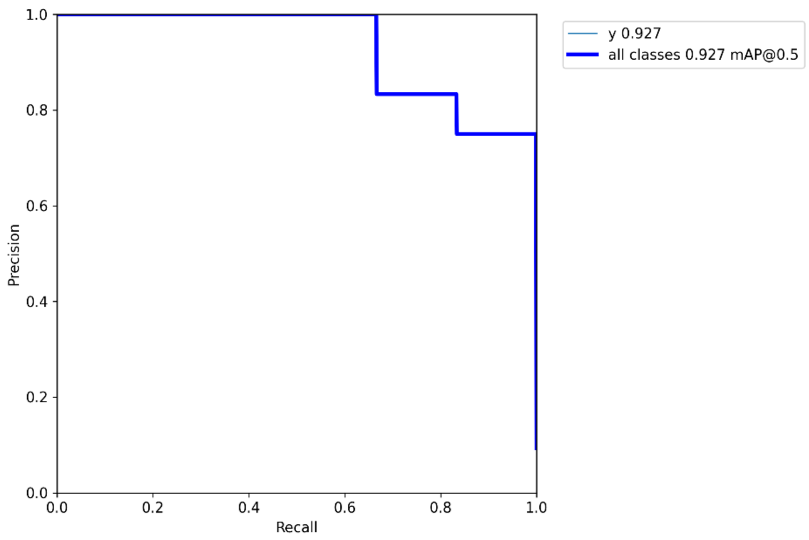

Supplemental Figure S11 The P-R Curve of DeepD&Cchl _E

Supplemental Figure S12

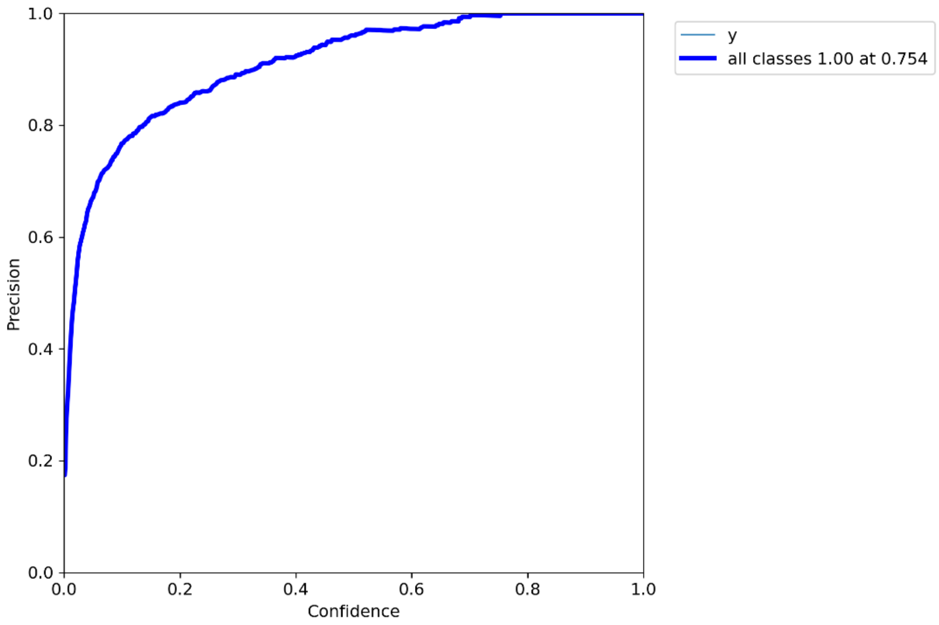

Supplemental Figure S12 The Precision Curve of DeepD&Cchl _F

Supplemental Figure S13

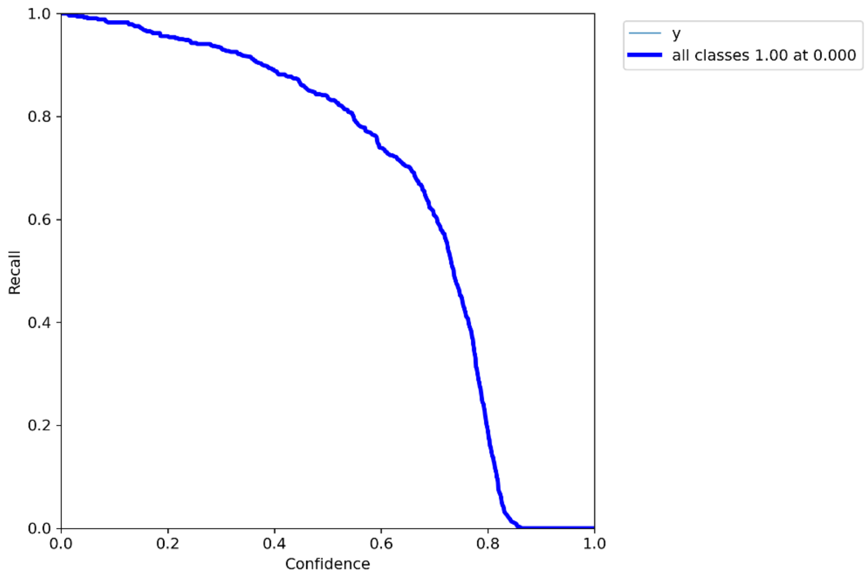

Supplemental Figure S13 The Recall Curve of DeepD&Cchl _F

Supplemental Figure S14

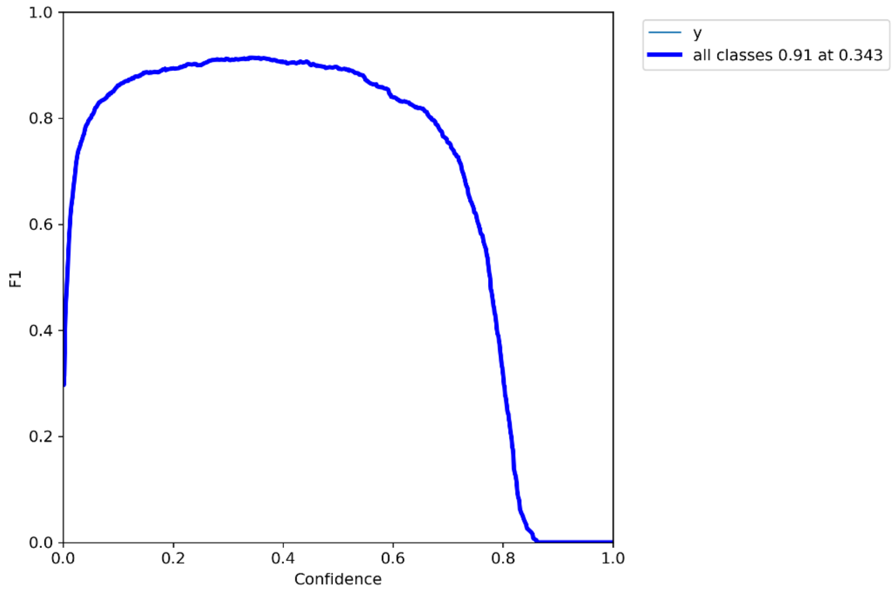

Supplemental Figure S14 The F1 Score Curve of DeepD&Cchl _F

Supplemental Figure S15

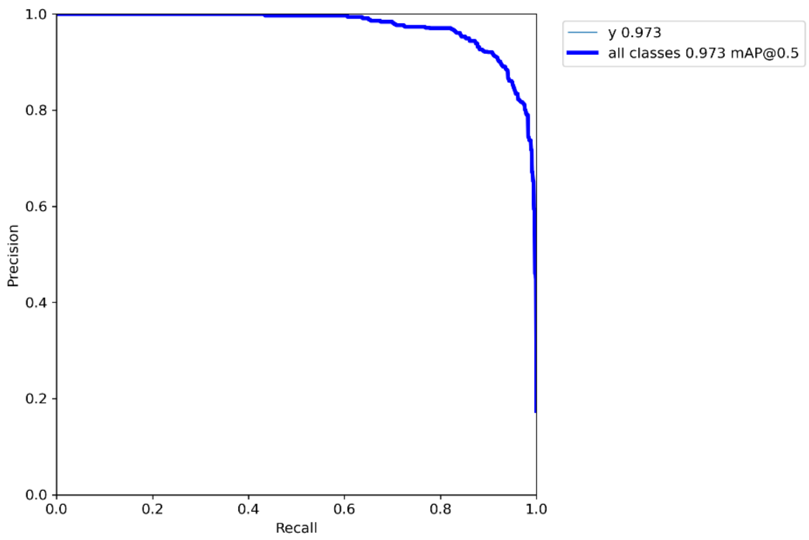

Supplemental Figure S15 The P-R Curve of DeepD&Cchl _F

Supplemental Figure S16

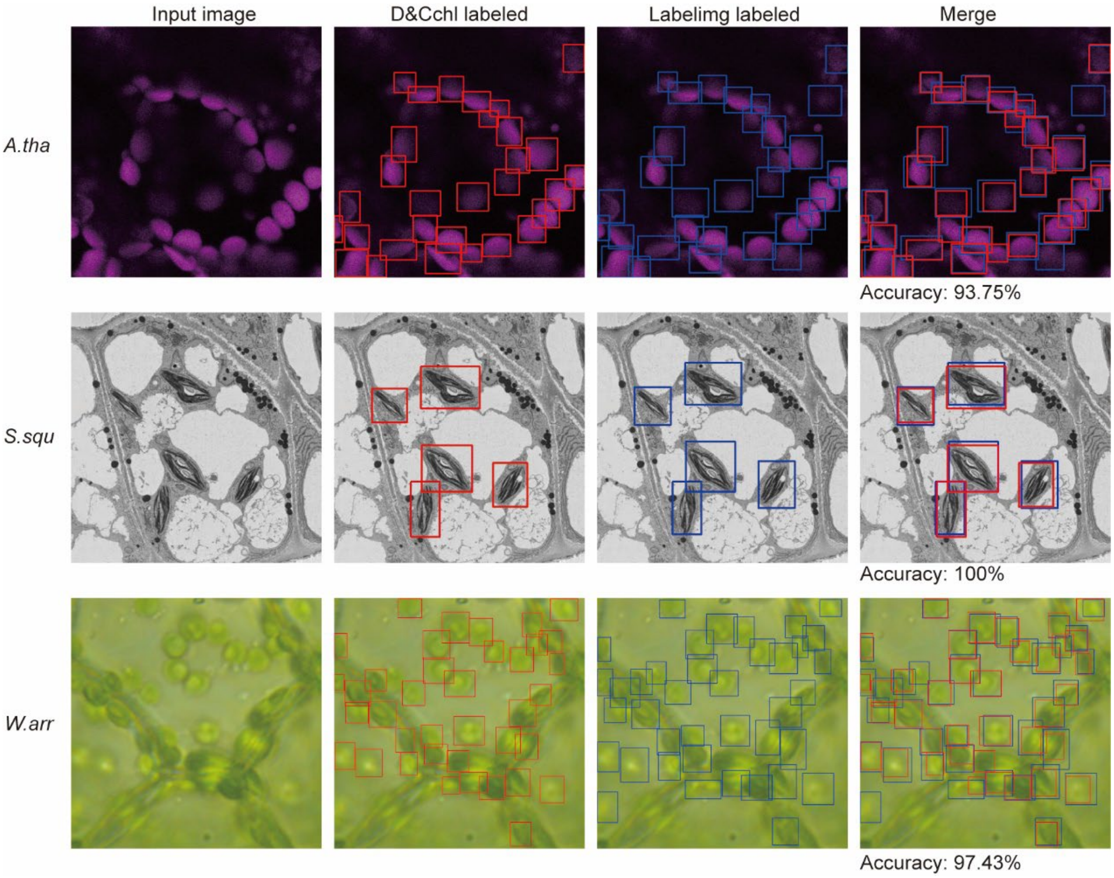

Supplemental Figure S16 Applications of DeepD&Cchl in detection of chloroplast across different image types

Chloroplasts detected are marked with red squares, while those identified manually are shown with blue squares. The study involves three types of images: fluorescent *Arabidopsis thaliana* [*A.tha*], electron microscopy images of *Sphagnum squarrosum* [*S.squ*], and *Wolffia arrhiza* [*W.arr*]. These images were tested using the DeepD&Cchl_F, DeepD&Cchl_E, and DeepD&Cchl_L models, respectively.

Supplemental Figure S17

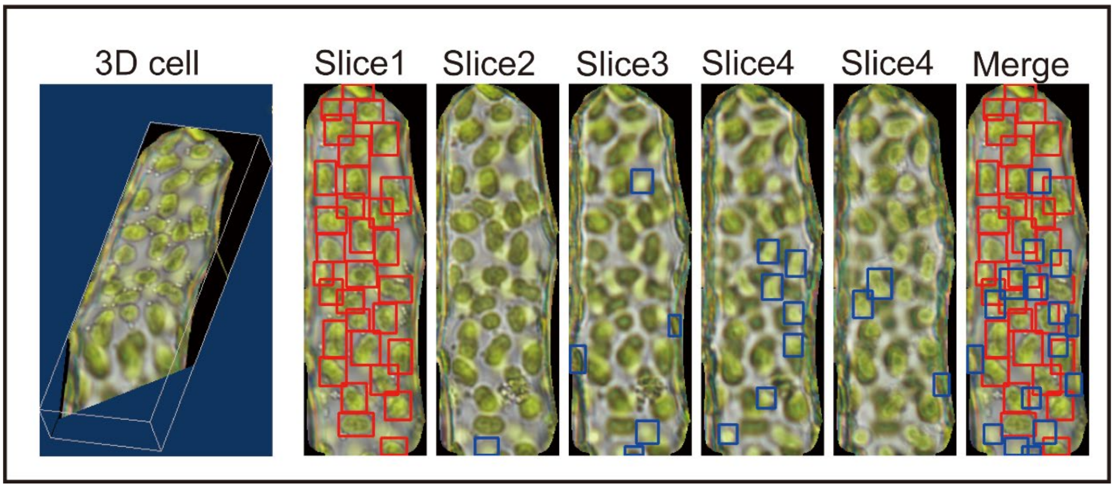

Supplemental Figure S17 3D single cell chloroplast detection using DeepD&Cchl

Slice 1 represents the first layer of selected images from the single-cell sample's microscopic image series, where all detected chloroplasts have been incorporated into the respective baseline. Slice 2 displays the results after excluding duplicate targets from the baseline. Subsequent images sequentially exhibit processed microscopic images and their chloroplast detection results, each showcasing new targets after successfully excluding previously identified duplicates from prior image slices. The final Merge layer consolidates results from all layers, with the first layer indicated by red boxes and subsequent layers indicated by blue boxes.

Supplemental Figure S18

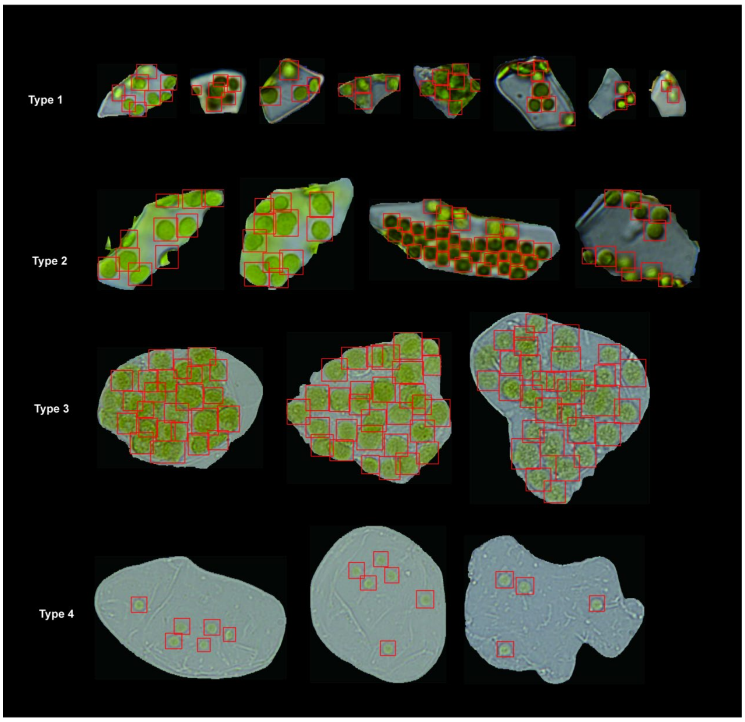

Supplemental Figure S18 Cell type specific single cell chloroplast detection using DeepD&Cchl

Examples of four types of single cells from Plants *Ricciocarpos natans* (*R. nat,* type 1 and type 2) and *Arabidopsis thaliana* (*A. tha,* type 3 and type 4).
